# Simultaneous Monitoring of Tyrosinase and ATP in Thick Brain Tissues Using a Single Two‐Photon Fluorescent Probe

**DOI:** 10.1002/advs.202413220

**Published:** 2025-03-24

**Authors:** Hong Huang, Huiru Li, Yong Zhang, Xuhan Xia, Ningwen Zhang, Haixin Fan, Longhua Guo, Yongyong Cao, Hu Pan, Ruijie Deng, Yangang Wang, Rodrigo Ledesma‐Amaro, Jianguo Xu

**Affiliations:** ^1^ College of Biological and Chemical Engineering Jiaxing University Jiaxing 314001 China; ^2^ College of Biomass Science and Engineering Sichuan University Chengdu 610065 China; ^3^ Department of Bioengineering, Imperial College Centre for Synthetic Biology Imperial College London London SW7 2AZ UK; ^4^ Engineering Research Center of Bio‐process Ministry of Education School of Food and Biological Engineering Hefei University of Technology Hefei 230009 China

**Keywords:** alzheimer's disease, ATP, brain tissues, two‐photon imaging, tyrosinase

## Abstract

Cellular redox homeostasis and energy metabolism in the central nervous system are associated with neurodegenerative diseases. However, their real‐time and concurrent monitoring in thick tissues remains challenging. Herein, a single dual‐emission two‐photon fluorescent probe (named **DST**) is designed for the simultaneous tracking of tyrosinase (TYR) and adenosine triphosphate (ATP), thereby enabling the real‐time monitoring of both neurocellular redox homeostasis and energy metabolism in brain tissue. The developed **DST** probe exhibits excellent sensitivity and selectivity toward TYR and ATP, with distinctive responses in the blue and red fluorescence channels being observed without spectra crosstalk. Using this probe, the correlation and regulatory mechanism between TYR and ATP during oxidative stress are uncovered. Additionally, the two‐photon nature of this probe allows alterations in the TYR and ATP levels to be monitored across different brain regions in an Alzheimer's disease (AD) mouse model. Notably, a significant decrease in ATP levels is revealed within the somatosensory cortex (S1BF) and caudate putamen brain regions of an AD mouse, alongside an increase in TYR levels within the S1BF and laterodorsal thalamic nucleus brain regions. These findings indicate the potential of applying the spatially resolved regulation of neurocellular redox homeostasis and energy metabolism to treat neurodegenerative diseases.

## Graphical abstract

A single dual‐emission two‐photon fluorescent probe is designed for simultaneously tracking tyrosinase (TYR) and adenosine triphosphate (ATP). With the help of this probe, the intricate correlation and regulatory mechanism between TYR and ATP during oxidative stress is uncovered. Furthermore, the two‐photon characteristic of this probe enables us to monitor fluctuations in the TYR and ATP levels across different brain regions.

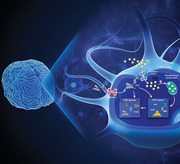

## 1 Introduction

Redox homeostasis and energy metabolism play key roles in the maintenance of neural function. An increasing number of studies have shown that in neurodegenerative diseases, such as Alzheimer's disease (AD) and Parkinson's disease (PD), neurons exhibit abnormal oxidative stress and impaired energy metabolism characteristics.^[^ ^1^ ^]^ Thus, investigating the molecules associated with redox homeostasis and energy metabolism not only provides insights into the pathological mechanisms of these diseases, but it may also offer new strategies for developing targeted therapeutic drugs. Tyrosinase (TYR) plays a pivotal role in the central nervous system (CNS), primarily regulating redox homeostasis of the CNS by catalyzing the oxidation of monophenols and o‐diphenols to their corresponding o‐quinones.^[^ ^2^ ^]^ The maintenance of redox homeostasis is often intricately linked with energy metabolism, in which adenosine triphosphate (ATP) plays key roles in its regulation.^[^ ^3^ ^]^ Therefore, the development of analytical tools to simultaneously monitor the dynamics of TYR and ATP in living cells is essential for investigating the intricate relationship between redox homeostasis and energy metabolism.

Due to its high spatial‐temporal resolution, real‐time visualization capability, and nondestructive nature, fluorescence imaging has become a powerful method for monitoring biological events in living cells and in vivo.^[^ ^4^ ^]^ To date, many small molecule fluorescent probes have been developed for the independent detection and imaging of TYR and ATP through different excitation modes, namely one‐photon and two‐photon excitation.^[^ ^5^ ^]^ Compared with one‐photon fluorescence imaging, two‐photon fluorescence imaging is particularly appealing due to its deep tissue penetration ability and high spatial resolution. Consequently, it has been employed for exploring the molecular events correlated with the neural redox homeostasis and energy metabolism.^[^ ^6^ ^]^ However, there is currently a lack of two‐photon fluorescent probes capable of performing the simultaneous and in situ detection of TYR and ATP in biosystems. Notably, if two fluorescent probes, each specifically responsive to either TYR or ATP, are employed concurrently, the intricate interplay between TYR and ATP in redox homeostasis and energy metabolism may remain unclear. This is due to the different uptake, distribution, and metabolism characteristics of the probes, which render it challenging to simultaneously detect TYR and ATP at the same site.^[^ ^7^ ^]^ As such, a novel fluorescent probe enabling the simultaneous and real‐time determination of TYR and ATP is required to uncover the correlation and regulatory mechanism between TYR and ATP.

**Scheme 1:**
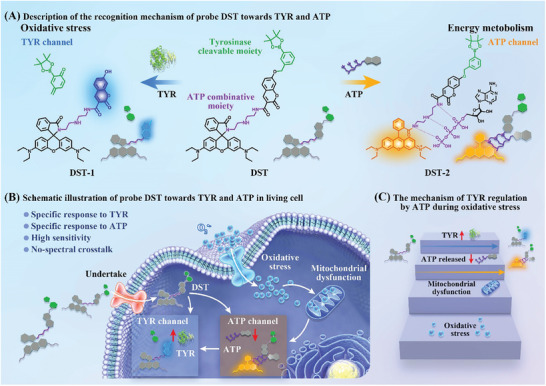
Schematic representations of the working principle of the developed **DST** probe. A) Recognition mechanism of the developed **DST** probe. B) Schematic illustration showing the simultaneous response of the **DST** probe toward TYR and ATP in a neuron. C) Mechanism of TYR regulation by ATP during oxidative stress.

Thus, we herein report the development of a single dual‐emission, two‐photon fluorescent probe (denoted **DST**) for the simultaneous determination of TYR and ATP in neurons, which is based on the intramolecular charge transfer–photoinduced electron transfer (ICT‐PET) mechanism (**Scheme** **1**A). More specifically, the fluorescent **DST** probe is applied for imaging and analyzing TYR and ATP within neurons. The effect of increasing the concentration of O_2_^•−^ or prolonging its stimulation time is investigated in terms of the ATP levels, and the corresponding effects on the oxidative homeostasis process in neurons and the TYR levels are determined (Scheme 1B,C). Furthermore, the **DST** probe is employed to monitor changes in the TYR and ATP levels in different brain regions in an AD mouse brain model. Overall, the aim of this study is to establish a fluorescence sensing molecular platform for the simultaneous detection of TYR and ATP via a dual‐emission channel at the level of neurons and brain tissue slices, which could be applicable for resolving the potential interrelationship between TYR and ATP in oxidative homeostasis and energy metabolism.

## 2 Results and Discussion

### 2.1 Development and Characterizations of the DST Probe

Scheme 1A shows the chemical structure of the **DST** probe and its specific reactions with both TYR and ATP. This probe was developed by integrating four functional domains, namely two fluorescent matrices (coumarin and rhodamine) and two recognition groups (phenyl‐borate and diethylenetriamine).^[^ ^8^ ^]^ Coumarin, a two‐photon fluorophore, was selected as one signal moiety and was conjugated with a TYR‐cleavable m‐benzene boronic acid pinacol ester (Bb) group,^[^ ^9^ ^]^ which acted as the TYR responsive unit. Due to the presence of the Bb group, the fluorescence of the coumarin fluorophore was inhibited. Upon the introduction of TYR, the Bb group was eliminated by TYR to form a hydroxycoumarin derivative (CM‐OH) and activate the ICT process, which led to an enhanced fluorescence intensity at 455 nm (F_455_).^[^ ^10^ ^]^ On the other hand, the complex formed between the diethylenetriamine and rhodamine moieties exhibited a PET effect due to the presence of a spirolactam ring, which resulted in total quenching of the two‐photon fluorescence of rhodamine. In the presence of ATP, ring‐opening of the spirolactam ring occurs due to the formation of hydrogen bonds between the multiple amino groups and phosphate groups of ATP.^[^ ^11^ ^]^ Accordingly, a significant fluorescence enhancement was observed at 588 nm (F_588_), which was well‐resolved from the emission of coumarin at 455 nm.

Consequently, the single two‐photon fluorescent **DST** probe, based on a single‐molecule design, was imparted with the ability to separately and simultaneously detect endogenous TYR and ATP. The condensation‐based synthetic procedure for probe preparation is shown in Scheme S1 (Supporting Information), and the characterization results are presented in Figures S1–S9 (Supporting Information).

### 2.2 Optical Properties of the DST toward TYR and ATP

**Figure 1:**
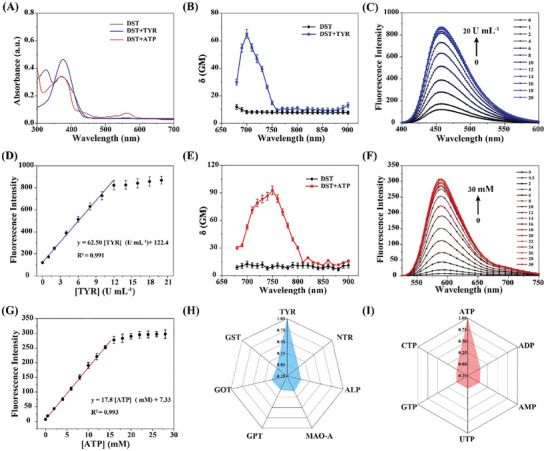
A) UV–vis absorption spectra of the **DST** probe before (black line) and after the addition of TYR (blue line) and ATP (red line). B) Two‐photon action cross‐section spectra of **DST** in the presence and absence of TYR. C) Two‐photon fluorescence spectra of the 5.0 µm **DST** probe in the presence of various TYR concentrations (0–20 U mL^−1^). D) Linear correlation between the TYR concentration and the fluorescence intensity at 455 nm. E) Two‐photon action cross‐section spectra of **DST** in the presence and absence of ATP. F) Two‐photon fluorescence spectra of the 5.0 µm **DST** probe in the presence of various ATP concentrations (0–30 mm). G) Linear correlation between the ATP concentration and the fluorescence intensity at 588 nm. Error bars represent the standard deviations (SD) for n = 10. H) Selectivity test performed for the 5.0 µm **DST** probe in the presence of various proteins, including NTR, ALP, MAO‐A, GPT, GOT, and GST (20 U mL^−1^ each). I) Selectivity test performed for the 5.0 µm **DST** probe in the presence of various energy‐relevant molecules, including ADP, AMP, UTP, GTP, and CTP (30 mm each). All data were recorded in PBS (10 mm, pH 7.4) containing 5% (v/v) DMSO using an excitation wavelength of 720 nm.

To validate **DST** as a dual‐color indicator for TYR and ATP, its optical response was examined in simulated physiological solutions. Initially, the absorption spectrum and two‐photon fluorescence titration spectra of **DST** were detected upon the addition of different concentrations of TYR in phosphate‐buffered saline (PBS). As shown in **Figure** **1**A, the absorption peak for **DST** at 380 nm increased 1.4‐fold upon the addition of TYR, indicating that the Bb group was cleaved by TYR. In addition, from the two‐photon absorption spectrum (Figure 1B), it can be seen that the addition of TYR caused the cross section (δ) value of **DST** to rise from 7.3 ± 1.3 to 57.2 ± 4.1 GM upon excitation at 720 nm. When the TYR concentration was increased from 0 to 20 U mL^−1^, the blue fluorescence of the F_455_ channel was gradually enhanced, reaching a plateau at TYR concentrations of 12 to 20 U mL^−1^ (Figure 1C). In particular, the addition of 12 U mL^−1^ TYR significantly enhanced the fluorescence intensity of the F_455_ channel, giving a value 6.8‐times greater than that observed in the absence of TYR. Furthermore, the fluorescence intensity of the F_455_ channel showed a good linear correlation with TYR concentration over a range of 0–12 U mL^−1^, giving the linear equation F_455_ = 62.50 [TYR] (U mL^−1^) + 122.4, with a linear correlation coefficient of 0.991 (Figure 1D). The limit of detection (LOD) for TYR was estimated to be 0.2 U mL^−1^ (3σ/K, where σ is the standard deviation of the blank sample, and K is the slope of the calibration curve).

Subsequently, the optical characteristics of the **DST** probe were evaluated in response to ATP. Upon the addition of ATP, a new absorption peak was observed at 563 nm, which could be attributed to the ATP‐induced opening of the spirolactam ring of rhodamine (Figure 1A). The two‐photon absorption spectrum (Figure 1E) showed that upon the addition of ATP, the δ value of **DST** increased from 11.6 ± 0.8 to 78.5 ± 3.2 GM under 720 nm excitation. In addition, the red fluorescence intensity of the F_588_ channel was found to increase dramatically upon increasing the ATP concentrations from 0 to 14 mm and then reached a plateau upon further increasing the ATP concentration from 14 to 30 mm (Figure 1F). More specifically, the addition of 14 mm ATP resulted in a significant 30‐fold enhancement in the F_588_ channel intensity, which showed a good linear correlation with ATP concentrations between 0 and 14 mm. The linear equation was defined as F_588_ = 17.8 [ATP] (mm) + 7.33, with a linear correlation coefficient of 0.993 (Figure 1G).

Evaluation of the probe stability was then performed, indicating that the two‐photon excited fluorescence demonstrated long‐term photostability and pH stability over a pH range of 5–9 (Figure S10, Supporting Information). In addition, the selectivity of the probe toward ATP and TYR was examined using potentially interfering proteins, such as nitroreductase (NTR), alkaline phosphatase (ALP), monoamine oxidase‐A (MAO‐A), glutamate pyruvate transaminase (GPT), glutamate oxaloacetate transaminase (GOT), glutathione S‐transferase (GST) (20 U mL^−1^ each). Similar selectivity evaluations were performed using various energy‐relevant molecules, including adenosine diphosphate (ADP), adenosine monophosphate (AMP), uridine triphosphate (UTP), guanosine triphosphate (GTP), and cytidine triphosphate (CTP) (30 mM each). As shown in Figure 1H,I, negligible fluorescence responses (<5.8%) were observed in the presence of these proteins and molecules. A series of competition studies were also performed (Figure S11, Supporting Information), and minimal impacts were found for the measurement of ATP and TYR.

### 2.3 Mechanistic Studies for the Molecular Interactions between DST and TYR/ATP

**Figure 2:**
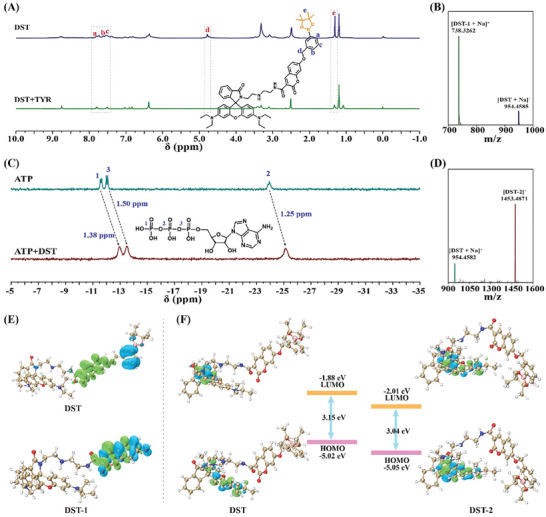
Mechanistic investigations into the molecular interactions between **DST**, TYR, and ATP. A) ^1^H NMR spectrum (D_2_O‐*d_2_*, 500 MHz) of **DST** (5 mm) in the presence of TYR (20 U mL^−1^). B) HR‐MS results for the **DST** probe in the presence of TYR. C) ^31^P NMR spectrum (D_2_O‐*d_2_*, 500 MHz) of **DST** (5 mm) in the presence of ATP (30 mm). D) HR‐MS results for the **DST** probe in the presence of ATP. E) Hole‐electron analysis of **DST** and **DST‐1**. F) HOMO‐LUMO energy levels of **DST** and **DST‐2**.

Mechanistic studies were performed to evaluate the molecular interactions between **DST**, TYR, and ATP. Initially, proton nuclear magnetic resonance (^1^H NMR) spectroscopy revealed that upon the addition of TYR to the **DST** probe, the signals corresponding to the 12 methyl H_c_ protons of the phenylboronic acid bis‐ethanolamine group essentially disappeared, as did the signals corresponding to the H_a_, H_b_, H_c_ of the phenyl, and that of the methylene H_d_ atom, thereby confirming removal of the TYR‐responsive group (Bb) (**Figure** **2**A).^[^ ^12^ ^]^ The structure of the product was further verified by high‐resolution mass spectrometry (HR‐MS, Figure 2B; Figure S12, Supporting Information), wherein the predominant ion detected at a mass to charge ratio (m/z value) of 738.3262 was attributed to [DST‐1+Na]^+^. In the case of ATP, ^31^P NMR spectroscopy was employed to investigate the molecular interactions. As shown in Figure 2C, the signals corresponding to the α, β, and γ phosphorus atoms underwent significant upfield shifts, shifting by 1.38, 1.50, and 1.25 ppm, respectively. These shifts were attributed to interactions between ATP and the **DST** probe, indicating that the probe binds to the α, β, and γ phosphate positions of ATP through hydrogen bonding.^[^ ^13^ ^]^ The structure of the product was further verified by HR‐MS (Figure 2D; Figure S13, Supporting Information), wherein the predominant ion detected at m/z 1453.4871 was attributed to [DST‐2]^+^.

The changes in luminescence upon the interaction of **DST** with TYR and ATP were investigated further by means of density functional theory (DFT) calculations. For the response between **DST** and TYR, the hole and electron distributions of both **DST** and **DST‐1** were evaluated. As shown in Figure 2E, the electrons of **DST** were primarily distributed across the coumarin backbone, while the holes were mainly on the Bb group. In contrast, for **DST‐1**, both electrons and holes were concentrated on the coumarin backbone, exhibiting typical ICT characteristics.^[^ ^14^ ^]^ Thus, after the interaction with TYR, elimination of the Bb group initiates the ICT process, significantly enhancing the fluorescence of the F_455_ channel. Similarly, the response between **DST** and ATP was evaluated using the frontline orbital theory, indicating that the highest occupied molecular orbital (HOMO) of **DST** was mainly distributed across the rhodamine backbone, while the lowest unoccupied molecular orbital (LUMO) was concentrated on the spirolactam ring. In the case of **DST‐2**, both the HOMO and the LUMO were localized on the rhodamine backbone, and these orbitals of **DST‐2** were both lower in energy than that of **DST** (Figure 2F). This indicates that following opening of the spirolactam ring upon the interaction of **DST** with ATP, the PET process between the rhodamine backbone and the spirolactam ring was inhibited, which led to the observed fluorescence increase in the F_588_ channel.

### 2.4 Biocompatibility and Cytotoxicity

Prior to employing **DST** for the imaging of TYR and ATP in neurons, its biocompatibility and cytotoxicity characteristics were evaluated. As shown in Figure S14 (Supporting Information), after culturing the neurons with the probe at concentrations of up to 50 µm for 24 h, negligible differences were observed between the control cells and those cultured with the probe. In addition, the MTT (3‐[4,5‐dimethylthiazol‐2‐yl]‐2,5 diphenyl tetrazolium bromide) assay was performed, which demonstrated that the neuron viability remained >90% when treated with **DST** at concentrations ranging from 0 to 30 µm for 24 h (Figure S15A, Supporting Information). These results confirm the high biocompatibility and negligible cytotoxicity of the **DST** probe.

### 2.5 Two‐Photon Fluorescence Imaging of TYR and ATP in Living Neurons

**Figure 3:**
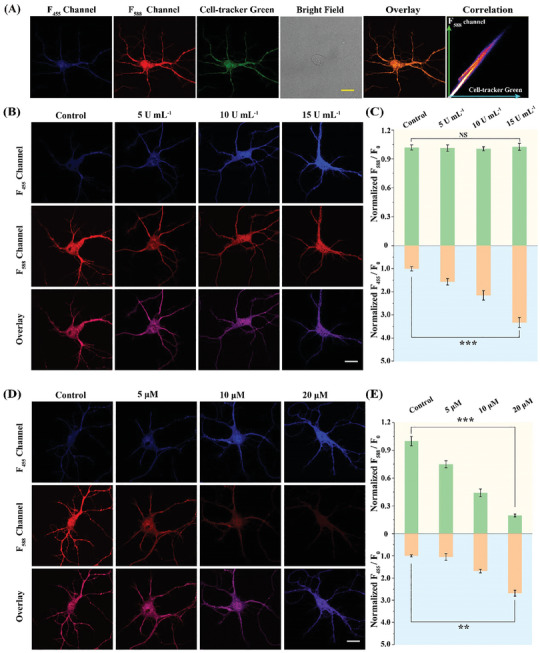
A) Fluorescence images of different channels of neurons incubated with **DST** (λ_ex_ = 720 nm; F_455_: λ_em_ = 400–480 nm, F_588_: λ_em_ = 520–620 nm) and CellTracker Green (λ_ex_ = 488 nm; λ_em_ = 500–580 nm). The overlay image was obtained from the F_588_ channel and CellTracker Green. B) Fluorescence imaging of neurons in the presence of TYR (0, 5, 10, and 15 U mL^−1^). C) Quantification of the fluorescent intensity ratio changes (F_455_/F_0_ and F_588_/F_0_) based on the data presented in panel (B). D) Fluorescence imaging of neurons in the presence of oligomycin (0, 5, 10, and 20 µm). E) Quantification of the fluorescent intensity ratio changes (F_455_/F_0_ and F_588_/F_0_) based on the data presented in panel (D). F_0_ represents the average fluorescence intensity from the control group. F_455_ and F_588_ denote the average fluorescence intensities from the experimental groups. Error bars indicate the SD for n = 5. Scale bar: 30 µm. Statistical significance was assessed using a two‐tailed unpaired t‐test, and the associated P values are specified in the figure legends as follows: ^NS^P > 0.05, ^**^ *p* < 0.01, and ^***^ *p* < 0.001.

Subsequently, the probe was simultaneously incubated with the commercially available cytoplasm probe CellTracker Green. As shown in **Figure** **3**A, the fluorescence signal of the F_588_ channel, which was ascribed to the reaction between **DST** and basal ATP, overlapped well with the signal of CellTracker Green, giving a Pearson's correlation coefficient of 0.95, and indicating that the **DST** probe effectively targeted the neuronal cytoplasm.^[^ ^15^ ^]^

Two‐photon fluorescence imaging experiments were then conducted for the simultaneous detection of TYR and ATP in the neurons. To stimulate the neurons, exogenous TYR at concentrations of 0, 5, 10, and 15 U mL^−1^ were added, and after 1 h of incubation, it was observed that the brightness of the F_455_ channel increased with a gradual increment in the TYR concentration (Figure 3B). Accordingly, at the highest TYR concentration of 15 U mL^−1^, the fluorescence intensity of the F_455_ channel was enhanced 3.3‐fold (Figure 3C). In contrast, the intensity of F_588_ channel remained relatively constant (Figure 3B,C).

The ability of the probe to monitor ATP within cells was then evaluated using the two‐photon mode. To stimulate the neurons, the intracellular ATP levels were modulated using oligomycin, a drug that inhibits FoF1‐ATPase and blocks ATP production.^[^ ^16^ ^]^ Upon increasing the concentration of oligomycin from 0 to 20 µm, a gradual decrease in the fluorescence intensity of the F_588_ channel was observed. Notably, upon the introduction of 5 µm oligomycin, the fluorescence intensity of the F_588_ channel dropped to ≈75% of the initial intensity. At a higher oligomycin concentration of 10 µm, the fluorescence intensity of the F_588_ channel decreased to ≈44%, accompanied by a 1.6‐fold enhancement in the fluorescence intensity of the F_455_ channel. A further increase in the oligomycin concentration to 20 µm resulted in the fluorescence intensity of the F_588_ channel reducing to ≈19% of its original value, while the fluorescence intensity of F_455_ channel increased 2.6‐fold (Figure 3D,E). These observations indicated that the **DST** probe is a reliable tool for concurrently assessing the fluctuations of TYR and ATP levels in neurons, wherein the synthesis of ATP regulates the level of TYR.

**Figure 4:**
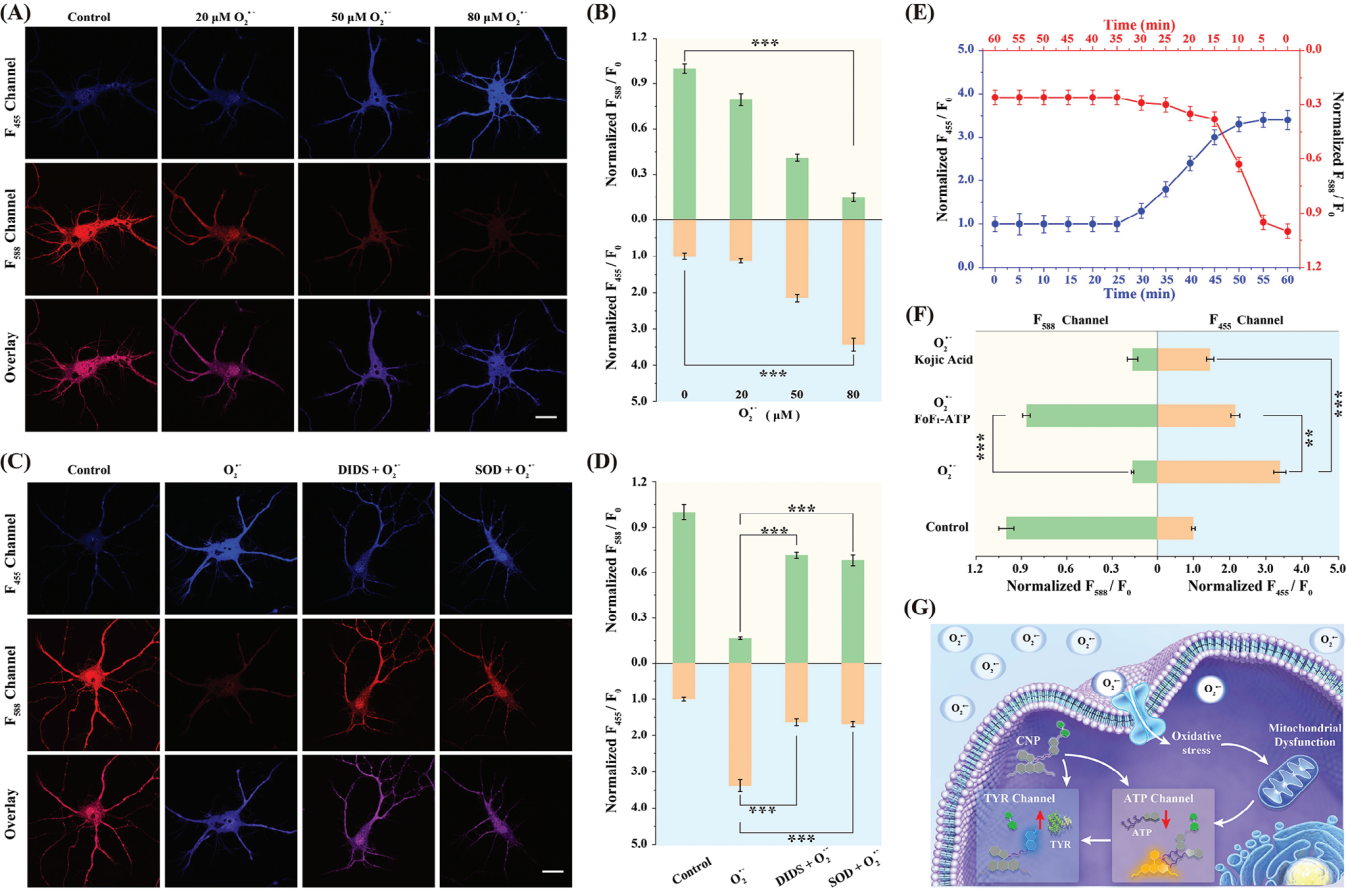
Two‐photon fluorescence imaging and real‐time quantification of TYR and ATP in neurons and brain tissues. A) Confocal microscopy imaging of the **DST** probe (5 µm) in the neurons under O_2_ **^•−^** (0, 20, 50, and 80 µm) stimulation. B) Quantification of fluorescent intensity ratio changes (F_455_/F_0_ and F_588_/F_0_) based on the results presented in panel (A). C) Confocal microscopy imaging of the neurons after treatment with 100 µm O_2_^•−^ in the presence of 150 µm DIDS or 100 U mL^−1^ SOD. D) Quantification of fluorescent intensity ratio (F_455_/F_0_ and F_588_/F_0_) based on the results presented in panel (C). E) Quantification of fluorescent intensity ratio changes (F_455_/F_0_ and F_588_/F_0_) under O_2_^•−^ stimulation at different times. F) Quantification of fluorescent intensity ratio changes (F_455_/F_0_ and F_588_/F_0_) after treatment with 100 µm O_2_^•−^ in the presence of 100 mg kg^−1^ kojic acid or 100 mg kg^−1^ F_0_F₁‐ATP. G) Illustration of the recognition mechanism of the **DST** probe in response to TYR and ATP in the neurons. Error bars represent the SD for n = 10. Scale bar: 30 µm.Asterisks indicate statistically significant changes (^**^ *p* < 0.01, ^***^ *p* < 0.001).

Then, the highly selective **DST** probe was employed to assess the impact of O_2_ **^•−^** induced changes in the neural TYR and ATP levels. It has been previously reported that a close correlation exists between O_2_ **^•−^**, the degree of intracellular oxidative stress, and energy metabolism.^[^ ^17^ ^]^ Thus, the impact of O_2_ **^•−^** on neurons was initially assessed via a cytotoxicity evaluation (Figure S15B), wherein it was found that the neuronal survival rate exhibited a considerable decline as the concentration of O_2_ **^•−^** was increased. In addition, it was observed that increasing the concentration of O_2_ **^•−^** from 0 to 80 µm resulted in a significant decrease in the fluorescence intensity of the F_588_ channel (**Figure** **4**A). Based on this result, the ATP level was calculated to have decreased to ≈15% of the initial level (Figure 4B). The findings suggested a dose‐dependent relationship between the level of ATP and the concentration of O_2_ **^•−^**. Furthermore, it was observed that upon the addition of 50 µm O_2_ **^•−^**, the ATP level decreased to ≈42% of its original level, and that was accompanied by a 2.1‐fold increase in the TYR level.

To confirm that the O_2_ **^•−^** species were responsible for these signal changes, an anion channel blocker (4,4′‐diisothiocyanatostilbene‐2,2′‐disulfonic acid disodium salt hydrate, DIDS, 150 µm) was used to precondition neurons to block O_2_ **^•−^** entry.^[^ ^13^, ^18^ ^]^ As a result, the addition of O_2_ **^•−^** induced only limited variations in the fluorescence intensities of the F_455_ and F_588_ channels. These results indicate that O_2_ **^•−^** could easily enter the neurons through the anion channels, causing oxidative damage and altering the ATP and TYR levels, and that such entry was blocked by the addition of DIDS (Figure 4C,D). Similar results were obtained upon pre‐treating the neurons with superoxide dismutase (SOD) (100 U mL^−1^). In addition, the results obtained for a Western blotting assay showed that the expression of TYR in neurons significantly increased after O_2_ **^•−^** stimulation, while TYR expression in neurons treated with DIDS decreased under the same conditions (Figure S16, Supporting Information). These data confirm that the fluorescence variations observed in the F_455_ channel under O_2_ **^•−^** stimulation were indeed due to the generation of TYR.

To investigate the imbalance of redox homeostasis and energy metabolism in neurons induced by O_2_ **^•−^**, the **DST** probe was used to test the stimulation of neurons at different time points using 100 µm O_2_ **^•−^**. As shown in Figure 4E and Figure S17 (Supporting Information), after 5 min of O_2_ **^•−^** stimulation, the ATP level declined rapidly in the neurons and plateaued within 10 min to reach ≈28% of the initial level. Simultaneous, it was observed that after 25 min of O_2_ **^•−^** stimulation, the TYR level in the neurons increased slowly, reaching a plateau within 20 min, and eventually increasing to 3.2‐fold the initial level. It was therefore postulated that O_2_ **^•−^** stimulation led to reduced ATP levels, which in turn further disrupted the redox balance and slowly increased the TYR level in the neurons.

Subsequently, to validate the signal pathway related to ATP regulation of the TYR levels, neurons were pre‐treated with kojic acid (a TYR inhibitor)^[^ ^5b^ ^]^ and F_0_F₁‐ATP (an ATP synthase agonist)^[^ ^19^ ^]^ prior to further stimulation of the neurons with 80 µm O_2_ **^•−^** for 30 min. It was observed that the addition of kojic acid to the neurons did not affect the ATP levels, whereas the level of TYR decreased to 1.5‐fold of the normal level.^[^ ^20^ ^]^ In contrast, for the neurons pre‐treated with F_0_F₁‐ATP, the ATP levels were restored to 87% of the normal level, and the level of TYR was 2.0‐fold higher than the normal level (Figure 4F; Figure S18, Supporting Information). These results clearly indicate that O_2_ **^•−^** stimulation induces abnormalities in neuron energy metabolism, promoting a significant reduction in the ATP levels, which then leads to a disruption of oxidative homeostasis and an increase in the TYR levels (Figure 4G).

### 2.6 Two‐Photon Imaging of the TYR and ATP Levels in AD Mouse Brain Tissues

**Figure 5:**
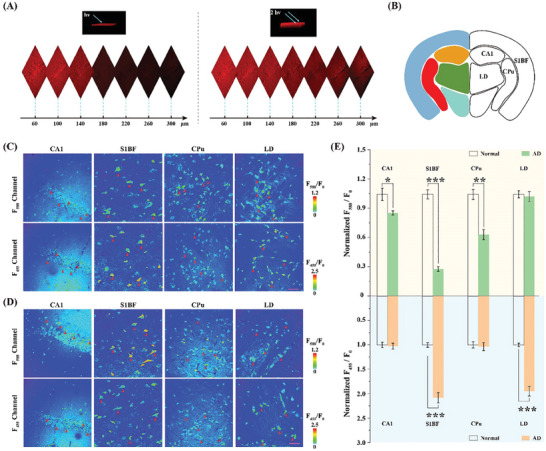
Two‐photon fluorescence imaging and real‐time quantification of TYR and ATP in mouse brain tissues. A) 3D one‐photon and two‐photon fluorescence images of the hippocampus region in the mouse brain labeled with **DST**, and excited at 405 and 720 nm, respectively. B) Illustration of the mouse brain regions (dark blue: cortex; red: striatum; green: thalamus; and yellow: hippocampus). C) Confocal microscopy imaging of TYR and ATP in the CA1, S1BF, CPu, and LD regions of the AD mouse brain slices. D) Confocal microscopy imaging of TYR and ATP in the CA1, S1BF, CPu, and LD regions of the normal mouse brain slices. Note: Neuron segmentation was performed to assess the fluorescence intensity contribution specifically from the neurons, rather than from the overall tissue. Red triangles indicate the representative neurons. E) Quantification of the fluorescent intensity ratio changes (F_455_/F_0_ and F_588_/F_0_) based on the data presented in panels (C) and (D). Error bars show the SD for n = 10. Scale bar: 75 µm. Asterisks indicate statistically significant changes (^*^ *p* < 0.05, ^**^ *p* < 0.01, and ^***^ *p* < 0.001).

Finally, the **DST** probe was employed to map TYR and ATP in four regions of AD and normal mouse brain tissues. As displayed in **Figure** **5**A, the two‐photon fluorescence image indicated deeper penetration (>300 µm, excitation at 720 nm) compared with the one‐photon image (≈180 µm, excitation at 405 nm). Subsequently, the **DST** probe (30 µm) was added to assess the levels of TYR and ATP in the brain tissues of the normal (C57BL/6) and AD (APP/PS1) mice.

More specifically, the **DST** probe was introduced to different regions of mouse brain slices, including the field CA1 of the hippocampus (CA1), the primary somatosensory cortex (S1BF), the caudate putamen (CPu), and the laterodorsal thalamic nucleus (LD), as shown in Figure 5B. As a result, the ATP levels in the AD mice decreased significantly in the S1BF and CPu regions compared with the normal mice (Figure 5C–E). In particular, a reduction to ≈28% of the original level was observed in the S1BF region, while a reduction to ≈60% was observed in the CPu region. In addition, the TYR levels in the AD mice increased dramatically in the S1BF and LD regions, i.e., by ≈2.1‐ and 1.9‐fold, respectively. These results suggest that the levels of TYR and ATP were heterogeneous across different regions of the AD mouse brain. Notably, memory loss is a hallmark symptom of AD, with the hippocampus and cortex being key regions involved in memory processing and likely being among the first areas affected by the disease.^[^ ^21^ ^]^ In AD mouse brain, the TYR levels in the cortical regions were found to be higher than those in the other regions, while the ATP levels were notably lower in these regions compared to other regions. These data indicated that the AD pathology has a pronounced impact on both the TYR and ATP levels in the cortical regions.

## 3 Conclusion

In this study, a fluorescence probe was developed to monitor redox homeostasis and energy metabolism in brains, both of which are important processes in the investigation of neural function and disease risk. Two fluorophores, namely coumarin and rhodamine, were selected for their large two‐photon absorption values and distinct emission profiles. Upon their modification with tyrosinase (TYR) and adenosine triphosphate (ATP) reactive moieties and subsequent conjugation through condensation, a small‐molecule probe (denoted **DST**) was obtained for the simultaneous monitoring of redox homeostasis and energy metabolism in living neurons and thick brain tissues.

Imaging of the TYR and ATP levels within O_2_ **^•−^** stimulated neurons unveiled the interplay between oxidative stress and energy metabolism in these cells, revealing a significant reduction in the ATP levels, and a corresponding substantial increase in the TYR levels. Taking advantage of the two‐photon characteristics of the **DST** probe, the levels of TYR and ATP were assessed in various brain regions of an Alzheimer's disease (AD) mouse brain model. Consequently, a significantly reduced ATP levels were observed in the primary somatosensory cortex (S1BF) and hippocampus regions, while elevated TYR levels were detected in the S1BF and laterodorsal thalamic nucleus regions compared to those measured for normal mice.

Overall, this work establishes a novel fluorescence sensing platform for the simultaneous detection of TYR and ATP at the cellular and brain tissue levels. This provides a valuable tool to investigate the molecular mechanisms underlying neural responses to oxidative stress. These findings not only advance our understanding of the AD pathology, but they also offer a new perspective for the development of novel treatments for neurodegenerative disease. Furthermore, this molecular design strategy has the potential for expansion to other biomolecules, including neurotransmitters, amino acids, and proteins.

## 4 Experimental Section

### Reagents and Chemicals

All chemicals were purchased from commercial suppliers and without further purification and modification. Rhodamine B, diethylenetriamine, adenosine triphosphate (ATP), triethylamine (NEt_3_), 7‐hydroxy‐2‐oxo‐2H‐chromene‐3‐carboxylic acid, 2‐(3‐(bromomethyl) phenyl)‐4,4,5,5‐tetramethyl‐1,3,2‐dioxaborolane, EDCI, HOBt were purchased from Adamas‐Beta Co., Ltd. (Shanghai, China). Ethanol (EtOH), dichloromethane (DCM), methanol (MeOH), and N, N‐dimethylformamide (DMF) were bought from General Reagents Co., Ltd. (Shanghai, China). Anhydrous Na_2_SO_4_, Na_2_S, NaCl, KCl, K_2_HPO_4_, KH_2_PO_4_, CaCl_2_, FeCl_2_, CuCl_2_, MgCl_2_·6H_2_O, Zn(NO_3_)_2_·6H_2_O, and H_2_O_2_ were purchased from Sinopharm Chemical Reagent Co., Ltd. (Shanghai, China). L‐histidine (His), tryptophan (Trp), L‐tyrosine (Tyr), dopamine (DA), nitroreductase (NTR), alkaline phosphatase (ALP), monoamine oxidase‐A (MAO‐A), glutamate pyruvate transaminase (GPT), glutamate oxaloacetate transaminase (GOT), glutathione S‐transferase (GST), adenosine diphosphate (ADP), adenosine monophosphate (AMP), uridine triphosphate (UTP), guanosine triphosphate (GTP), cytidine triphosphate (CTP), epinephrine (Ep), and norepinephrine (NE) were purchased from Aladdin Chemistry Co. Ltd. (Shanghai, China). CellTracker Green was purchased from Thermo Fisher Scientific (Invitrogen). Superoxide anion (O_2_ **^•−^**) was prepared from a classical XA/XOD system. Its concentration was determined by measuring the reduction of ferricytochrome c (ε_550_ = 21.1 mM^−1^ cm^−1^) spectrophotometrically using an UV–vis spectrophotometer. Hydroxyl radical (•OH) was derived from Fenton reaction (Fe^2+^/H_2_O_2_ = 1:5). Peroxynitrite (ONOO^–^) was generated by the reaction of NaNO_2_ with H_2_O_2_. H_2_S and HClO were derived from Na_2_S and NaClO, respectively. The concentration of O_2_ **^•−^**, ONOO^−^, H_2_O_2_, and HClO were obtained by the UV–vis absorption spectrophotometry. Phosphate buffer solution (PBS, pH 7.4) with concentration of 0.1 m was prepared from KH_2_PO_4_, K_2_HPO_4_·3H_2_O, and KCl.

### Instruments

The ^1^H NMR and ^13^C NMR spectra were obtained from a 500 MHz Bruker NMR spectrometer (Bruker, Germany). The mass spectra were detected by a Bruker ESI time‐of‐flight MS system (Bruker, Germany). The fluorescence spectrum and the UV–vis absorption spectrum was recorded by using a Hitachi F‐7000 fluorescence spectrometer (Hitachi, Japan) and a Hitachi UH‐5300 spectrometer (Hitachi, Japan), respectively. The fluorescence imaging was obtained from a Leica TCS SP8 confocal laser scanning microscope (Leica, Germany) equipped with two‐photon laser (Chameleon Ultra II, Coherent, UK). Two‐photon fluorescence images of the **DST** probe in neurons or brain slices were collected in the wavelength range of 400–480 nm (F_455_ channel) and 520–620 nm (F_588_ channel) under 720 nm excitation (output power: 2670 mW; detector gain: 40%). As for the one‐photon fluorescence images of CellTracker Green, the excitation wavelength was set at 488 nm, and the fluorescence emission signal was collected in a wavelength range of 500–580 nm. The cytotoxicity assays were measured by Varioskan LUX multimode microplate reader (Thermo Fisher scientific, USA). The apoptosis assay was carried out by a FACS Calibur flow cytometry (Becton, Dickinson and Company, USA). The fresh mouse brain tissue slices were obtained using a Leica VT3000 vibrating‐blade microtome (Germany) with a thickness of ≈300 µm.

### Cell Culture

The acquisition and cultivation of neurons were conducted as a previously reported procedure.^[^ ^22^ ^]^ Newborn within 24 h C57BL/6 wild‐type mice were anesthetized by halothane, and then their brains were removed quickly and put in Hanks’ balanced salt solution (HBSS, free Mg^2+^ and Ca^2+^) at 0 °C. Tissues of the cortex were stripped out and then incubated with papain at 37 °C for 12 min, after that they were dispersed into poly‐d‐lysine‐coated 35 mm Petri dishes at a density of 1 ×10^6^ cells/dish. The neurons were cultured with neurobasal medium containing L‐glutamine and B27 and the medium was changed three times a week. After maintained at 37 °C in a humidified atmosphere with 5% CO_2_ incubator for a week, the neurons could be used for imaging.

### Cytotoxicity and Apoptosis Assay

The cytotoxicity assays were measured by 3‐(4,5‐dimethylthiazol‐2‐yl)‐2,5‐diphenyltetrazolium bromide (MTT). Neurons in 96‐well plates were incubated with different concentrations of the **DST** probe (0, 5, 10, 15, 20, and 30 µm) and cultured for 12 and 24 h. Then, the neurons in each well were treated with 20 µL MTT solution (5 mg mL^−1^) and continuously incubated for 4 h at 37 °C. After that, MTT solution was removed and 100 µL DMSO was added to each well until the crystalline formazan products were dissolved. Absorbance was next measured at 490 nm in a Varioskan LUX multimode microplate reader (Thermo Fisher scientific, USA). Cell viability was defined as the ratio of absorbance in the experimental groups to that in the blank control groups. For apoptosis assays, the Annexin V‐FITC Apoptosis Detection Kit was used to determine the degree of cell apoptosis. Neurons were incubated with the **DST** probe (0, 10, 30, and 50 µm) for 24 h, then they were collected with the help of EDTA‐free trypsin and washed by 5 mL PBS for three times. Moreover, PBS was removed by centrifugation of 1000 rpm for 5 min and neurons were incubated with 195 µL binding buffer of Annexin V‐FITC, 5 µL Annexin V‐FITC, and 10 µL propidium iodide (PI) at room temperature in the dark for 30 min. After these procedures, neurons were used for the flow cytometry and detected at an excitation wavelength of 488 nm.

### Preparation and Imaging of Mouse Brain Tissue Slices

All animal experiments were carried out in strict adherence to the guidelines of the Care and Use of Laboratory Animals formulated by the Ministry of Science and Technology of China and were approved by the Animal Care and Use Committee of East China Normal University (approval number: m+ R20190304, Shanghai, China). Five‐month‐old normal mice and AD mice were purchased from the Laboratory Animal Center of the Chinese Academy of Science. The fresh mouse brain tissue slices with a thickness of ≈300 µm were obtained using a Leica VT3000 vibrating‐blade microtome (Germany). This procedure was meticulously performed in ice cold artificial cerebrospinal fluid (ACSF), composed of 124.0 mm NaCl, 3.0 mm KCl, 26.0 mm NaHCO_3_, 1.24 mm NaH_2_PO_4_, 8.0 mm MgSO_4_, 0.1 mm CaCl_2_, and 10.0 mm D‐glucose under a 95% O_2_ and 5% CO_2_ atmosphere. Subsequently, the slices were transferred to an incubation chamber filled with ACSF containing 30 µm **DST** and maintained at 37 °C for 60 min. The ACSF in the chamber was aerated with 95% O_2_ and 5% CO_2_. Following incubation, the treated slices were then thoroughly washed with ACSF at least three times prior to imaging. Finally, the stained slices were imaged using a TCS‐SP8 confocal laser‐scanning microscope equipped with a multiple‐photon laser, excited at 720 nm.

### Statistical Analysis

Data were obtained from at least three independent measurements (n ≥ 3). All the data are presented as means ± SD (standard deviation) unless otherwise indicated. Statistical significance was assessed using a two‐tailed unpaired t‐test, and the associated P values are represented as ^NS^P > 0.05 (NS: not significant), ^*^ *p* < 0.05, ^**^ *p* < 0.01, and ^***^ *p* < 0.001.

## Conflict of Interest

The authors declare no conflict of interest.

## Supporting information



Supporting Information

## Data Availability

The data that support the findings of this study are available on request from the corresponding author. The data are not publicly available due to privacy or ethical restrictions.
