# Supplementary material for: Simultaneous Monitoring of Tyrosinase and ATP in Thick Brain Tissues Using a Single Two‐Photon Fluorescent Probe
Source: Adv Sci (Weinh). 2025 Mar 24;12(19):2413220. doi: 10.1002/advs.202413220 (PMC12097068; doi:10.1002/advs.202413220)
Supplement: Supplementary file 1 — Supporting Information [file ADVS-12-2413220-s001.docx]

Supporting Information

Simultaneous Monitoring of Tyrosinase and ATP in Thick Brain Tissues Using a Single Two-Photon Fluorescent Probe

Hong Huang^1^, Huiru Li^1^, Yong Zhang^2^, Xuhan Xia^2^, Ningwen Zhang^1^, Haixin Fan^1^, Longhua Guo^1^, Yongyong Cao^1^, Hu Pan^1^, Ruijie Deng^2,*^, Yangang Wang^1,*^, Rodrigo Ledesma-Amaro^3^, and Jianguo Xu^1, 4*^

**Contents**

1. Synthesis and characterization of **DST**.

2. Stabilities of **DST** probe toward determination of TYR and ATP.

3. Competition tests of **DST** probe toward determination of TYR and ATP.

4. Sensing mechanism of **DST** probe to TYR and ATP.

5. FACS and MTT measurements of **DST** probe in live cells.

6. Western blot assay.

7. Two-photon microscope imaging of TYR and ATP in response to O_2_^•−^ stimulation.

# Synthesis and characterization of DST.


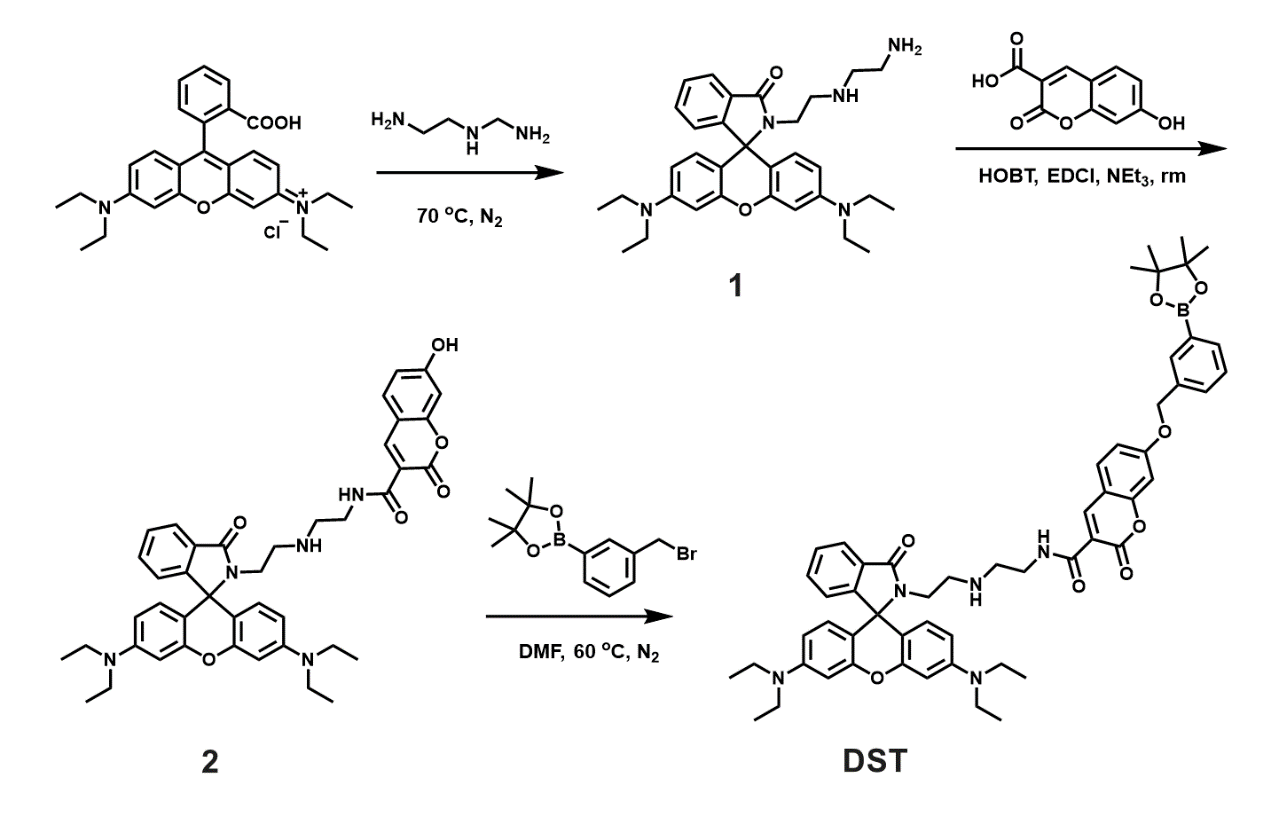


**Scheme S1**. The synthesis procedures of **DST**.

**Synthesis of Compound 1:** To a solution of rhodamine B (110 mg, 0.23 mmol) in EtOH (20 mL) was added diethylenetriamine (575 μL, 2.3 mmol). The solution was refluxed in N_2_ atmosphere for 3 h at 70 °C until color is disappeared. The solvent was evaporated under reduced pressure, and the reaction mixture was partitioned between a saturated NaHCO_3_ solution and DCM, and then the organic layer was separated. The aqueous layer was further extracted twice with DCM, and the combined organic extracts were washed with brine, dried over anhydrous Na_2_SO_4_, and the solvent was evaporated under reduced pressure. The crude product was purified by column chromatography on silica gel (eluent: MeOH:DCM:NEt_3_ = 20:100:1, v/v/v) to obtain compound **1** as a yellow solid. The ^1^H NMR, ^13^C NMR and HR-MS spectra of compound **1** are shown in Figures S1-S3, respectively. ^1^H-NMR (500 MHz, 298 K, DMSO-*d*_6_): δ 7.79-7.76 (m, 1H), 7.50-7.46 (m, 1H), 7.01-6.99 (m, 1H), 6.38-6.31 (m, 6H), 3.33-3.29 (m, 8H), 3.18 (s, 2H), 3.07-3.04 (m, 2H), 2.41-2.38 (m, 2H), 2.24-2.18 (m, 4H), 1.09-1.06 (m, 12H). ^13^C-NMR (125 MHz, 298 K, DMSO-*d*_6_): δ 167.4, 153.8, 153.1, 148.8, 133.0, 131.1, 128.8, 124.0, 122.7, 108.6, 105.6, 97.7, 64.5, 52.0, 49.0, 44.1, 41.6, 12.8. HR-ESI-MS m/z: [M+Na]^+^ calcd for [C_32_H_41_N_5_NaO_2_]^+^, 550.3152; found, 550.3150.


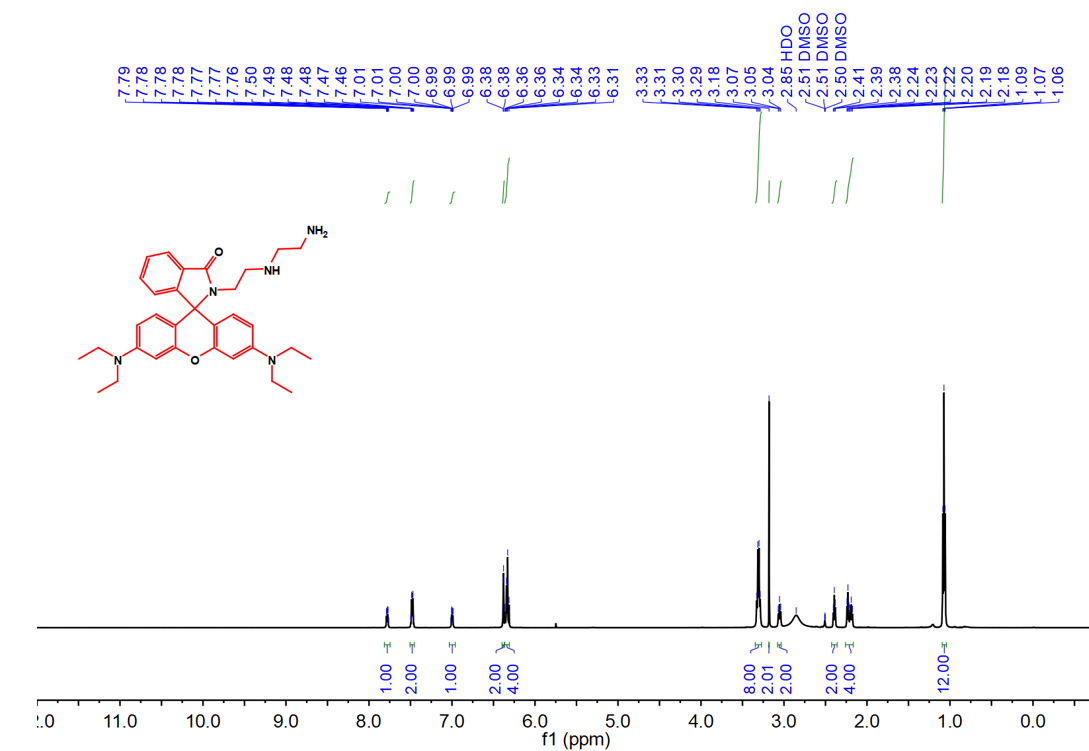


**Figure S1**. ^1^H NMR spectrum of compound **1**.


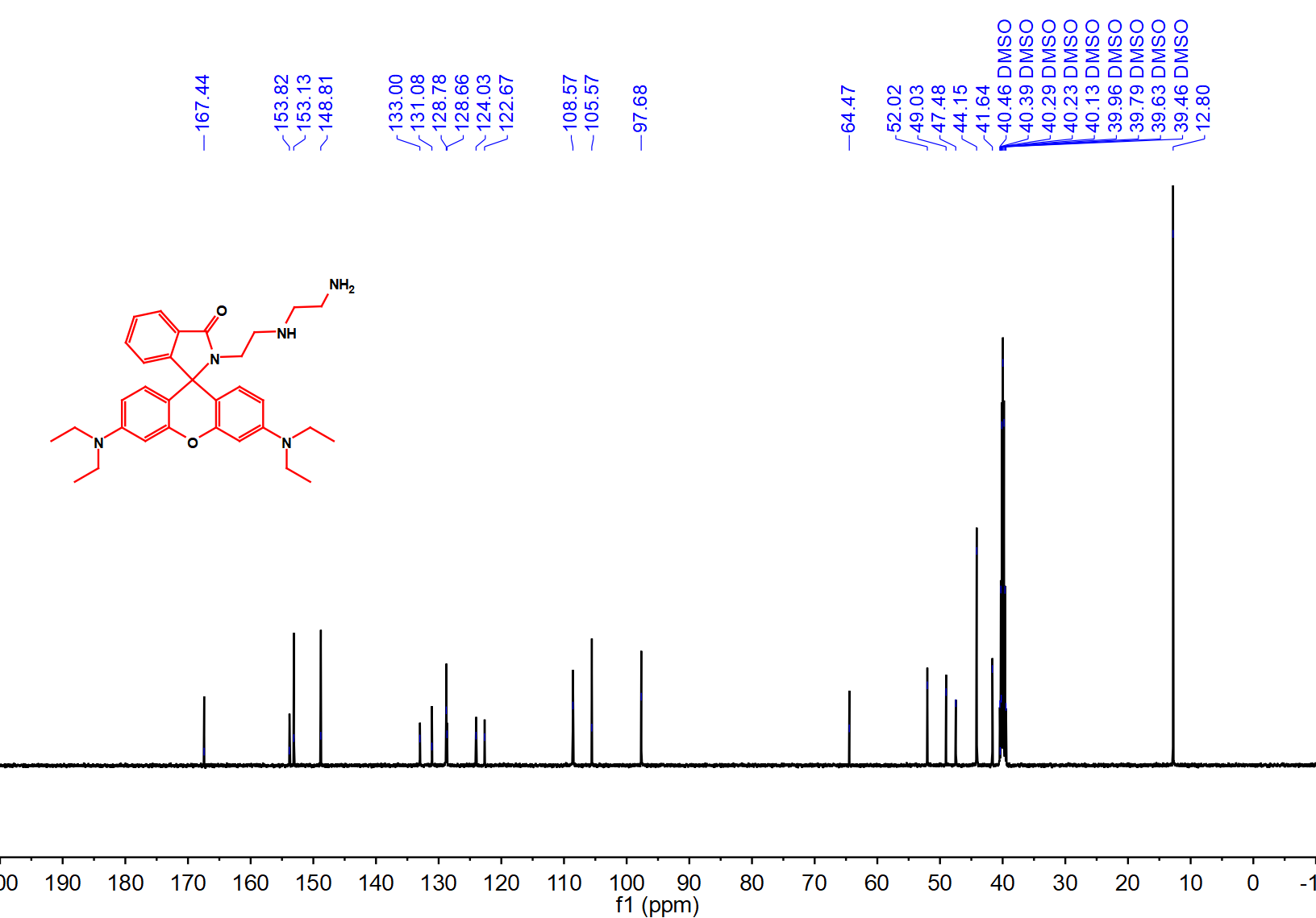


**Figure S2**. ^13^C NMR spectrum of compound **1**.


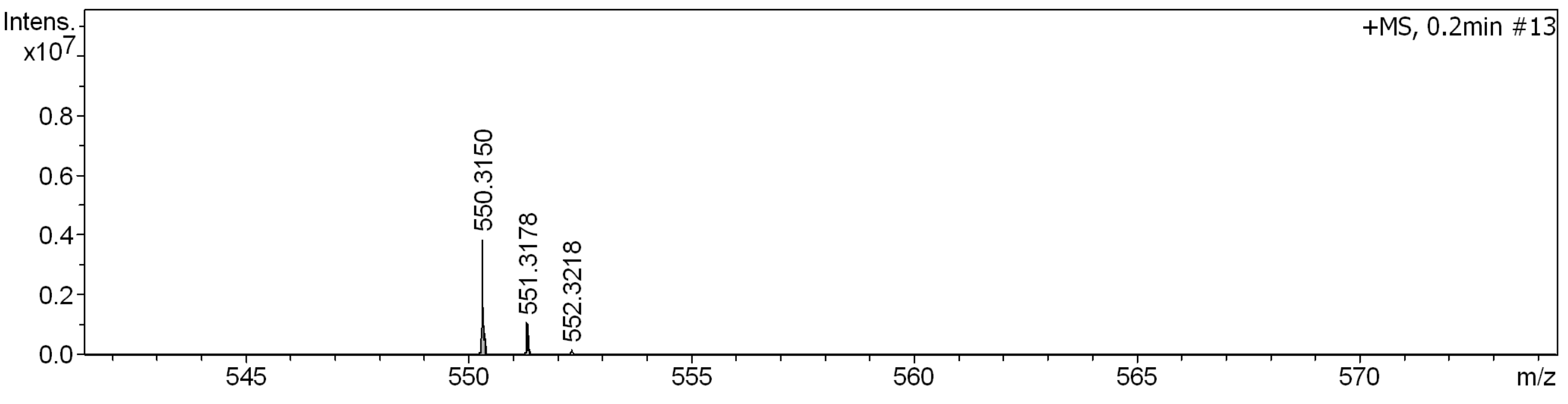


**Figure S3**. HR-MS spectrum of compound **1**.

**Synthesis of Compound 2:** Dissolve the Compound **1** (527 mg, 1 mmol) and 7-hydroxy-2-oxo-2H-chromene-3-carboxylic acid (290 mg, 1.4 mmol) in DCM solution, then HOBT (190 mg, 1.4 mmol), EDCI (382 mg, 2 mmol) and NEt_3_ (0.8 ml, 4 mmol) were added in the mixed solution, stirred the mixture for 24 h at room temperature. Then, the reaction mixture was concentrated under vacuum, and the crude product was purified by silica column chromatography (eluent: DCM:MeOH = 200:1 to 20:1, v/v) to obtain compound **2**. The ^1^H NMR,^13^C NMR and HR-MS spectra of compound **2** are shown in Figures S4-S6, respectively. ^1^H NMR (500 MHz, 298 K, DMSO-*d_6_*): δ 8.74-8.72 (m, 2H), 7.80-7.76 (m, 2H), 7.53-7.47 (m, 2H), 7.03-7.01 (d, *J* = 7.0 Hz, 1H), 6.91-6.89 (dd, *J_1_* = 2.0 Hz, *J_2_* = 8.5 Hz, 1H), 6.83-6.82 (d, *J* = 9.0 Hz, 1H), 6.40-6.35 (m, 6H), 3.35-3.21 (m, 10H), 3.20-3.18 (m, 2H), 3.05-3.02 (m, 2H), 2.69-2.67 (m, 2H), 2.44-2.42 (m, 2H), 1.08-1.06 (m, 12H). ^13^C NMR (125 MHz, 298 K, DMSO-*d_6_*): δ 167.9, 164.6, 162.4, 161.3, 156.8, 153.9, 148.9, 132.4, 128.7, 124.1, 122.8, 115.0, 113.6, 111.3, 108.6, 105.1, 102.3, 97.7, 64.6, 47.6, 46.6, 45.9, 44.1, 12.8, 9.1. HR-ESI-MS m/z: [M+Na]^+^ calcd for [C_42_H_45_N_5_NaO_6_]^+^, 738.3262; found, 738.3268.


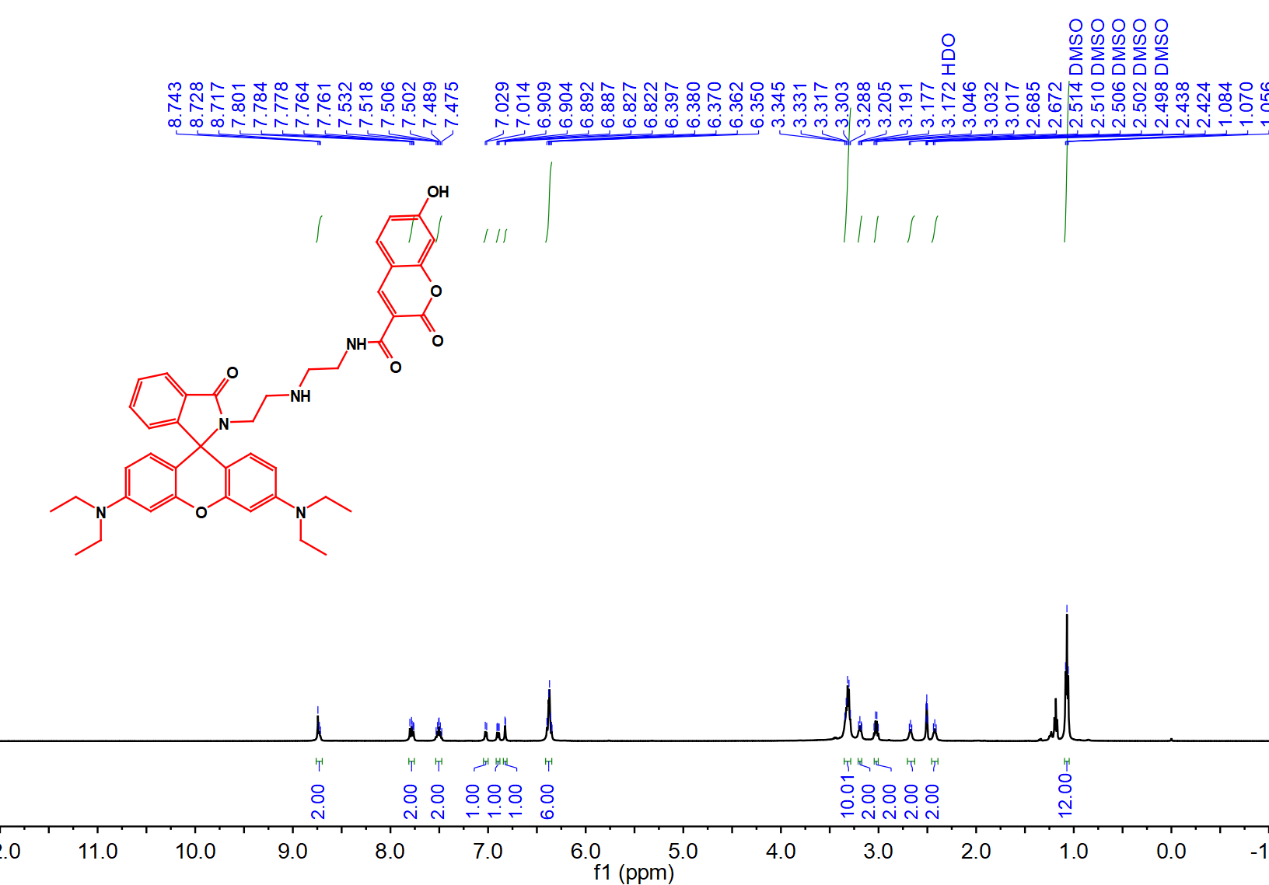


**Figure S4**. ^1^H NMR spectrum of compound **2**.


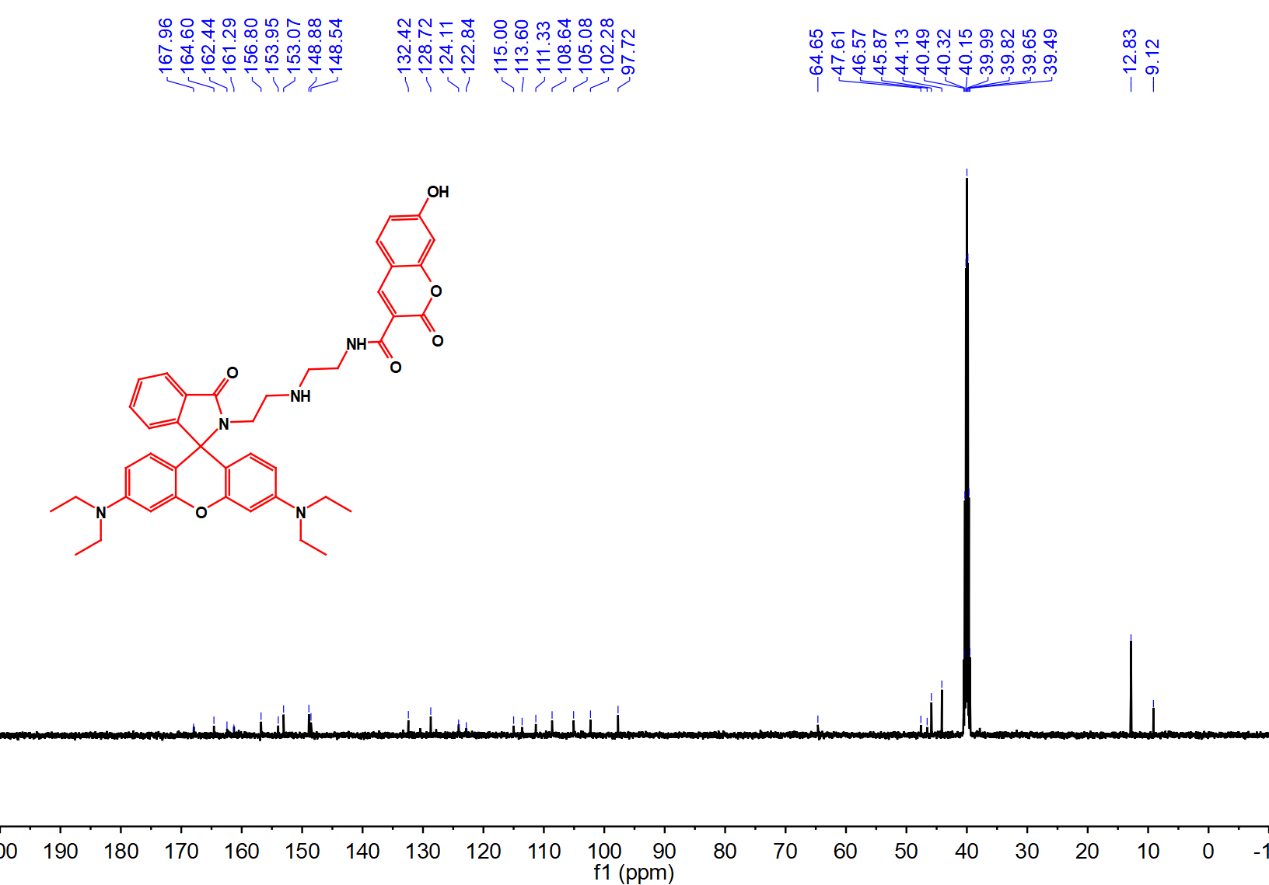


**Figure S5**. ^13^C NMR spectrum of compound **2**.


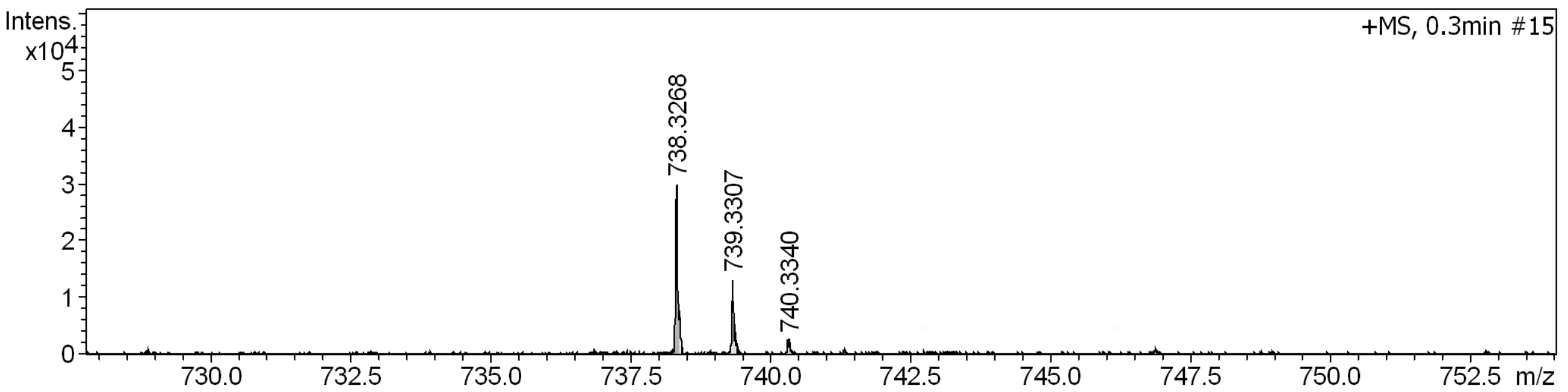


**Figure S6**. HR-MS spectrum of **Compound 2**.

**Synthesis of DST:** Dissolve the Compound **2** (715 mg, 1 mmol) and 2-(3-(bromomethyl) phenyl)-4,4,5,5-tetramethyl-1,3,2-dioxaborolane (415 mg, 1.4 mmol) in anhydrous DMF of 20 mL. Heat the reactant solution to 60 °C and stir for 12 h under N_2_ atmosphere. Cool the mixture to room temperature and remove the solvent by reduced pressure distillation. Then, the reaction mixture was concentrated under vacuum, and the crude product was purified by silica column chromatography (eluent: DCM:MeOH = 200:1 to 50:1, v/v) to obtain compound **DST**. The ^1^H NMR, ^13^C NMR and HR-MS spectra of compound **DST** are shown in Figures S7-S9, respectively. ^1^H NMR (500 MHz, 298 K, DMSO-*d_6_*): δ 8.82-8.76 (m, 2H), 8.43 (s, 2H), 7.82-7.74 (m, 4H), 7.41-7.38 (m, 2H), 7.03 (s, 1H), 6.91-6.89 (d, *J* = 8.5 Hz, 1H), 6.83 (s, 1H), 6.44-6.38 (m, 6H), 4.79 (s, 2H), 3.35-3.30 (m, 10H), 3.10-2.50 (m, 8H), 1.31-1.18 (m, 12H), 1.08-1.05 (m, 12H). ^13^C NMR (125 MHz, 298 K, DMSO-*d_6_*): δ 166.8, 163.3, 159.8, 158.4, 156.4, 154.9, 153.1, 149.4, 149.0, 135.7, 135.3, 134.7, 132.8, 132.3, 128.7, 117.8, 115.6, 113.1, 108.7, 104.6, 102.4, 97.7, 84.3, 64.9, 46.5, 46.2, 44.1, 42.0, 34.8, 25.1, 12.8, 9.1. HR-ESI-MS m/z: [M+H]^+^ calcd for [C_55_H_66_BN_5_O_8_]^+^, 932.4770; found, 932.4774.


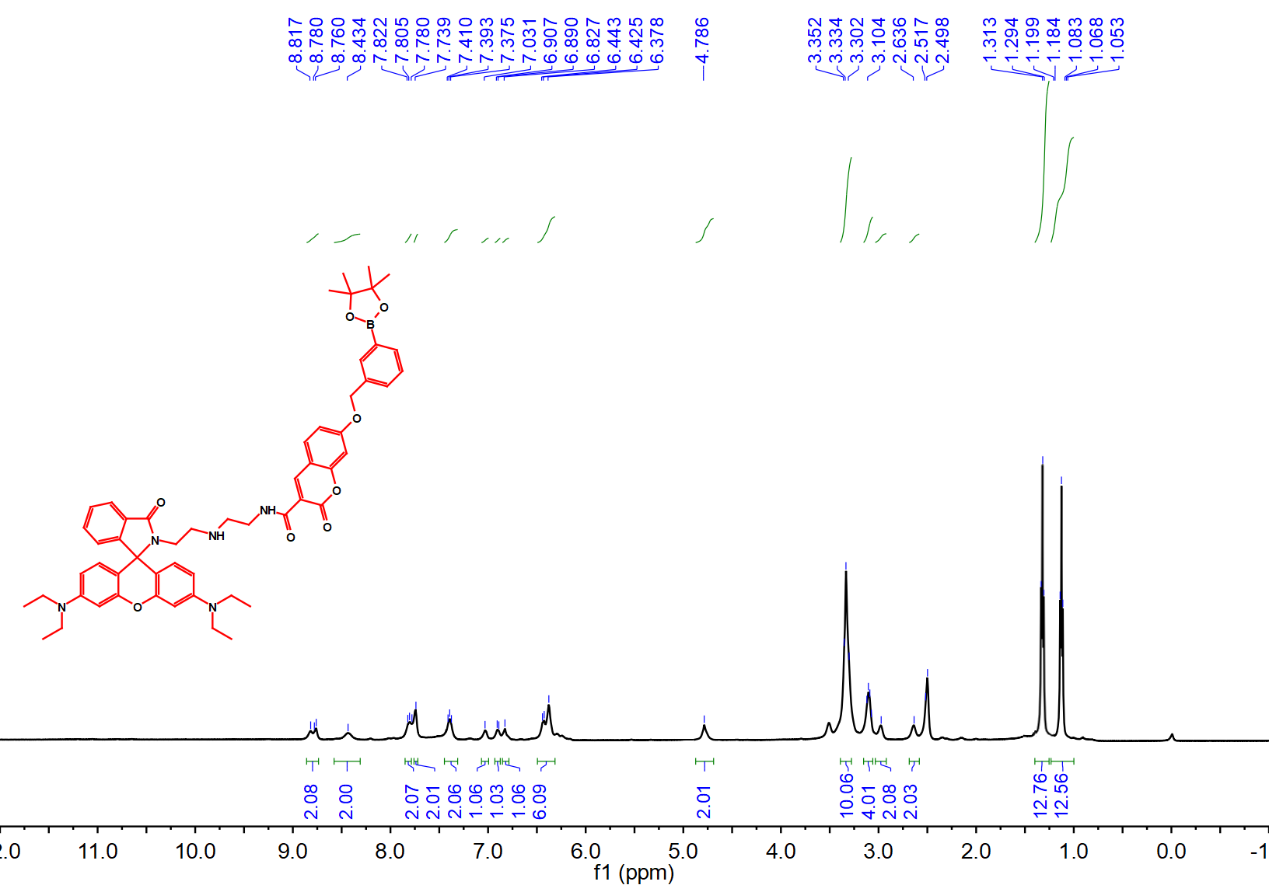


**Figure S7**. ^1^H NMR spectrum of **DST**.


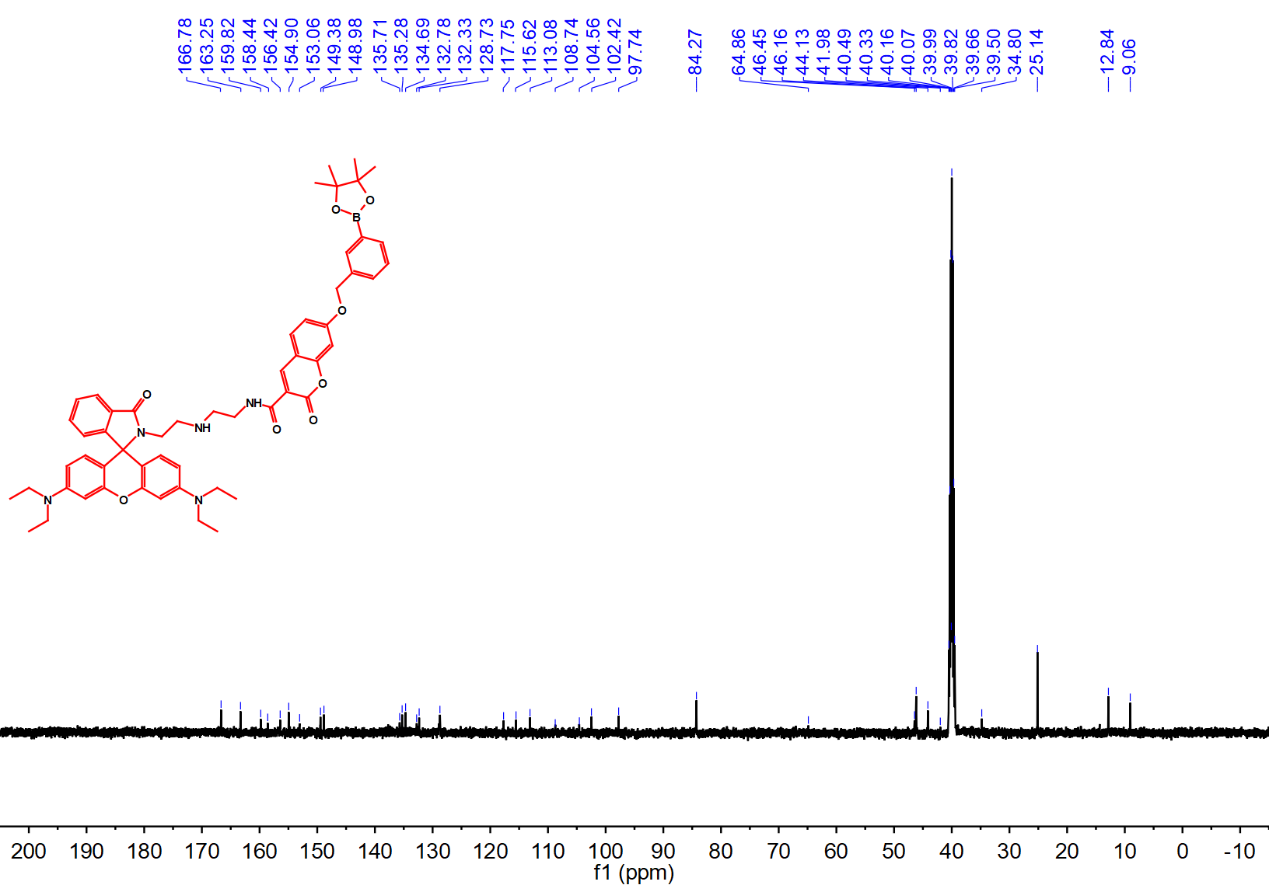


**Figure S8**. ^13^C NMR spectrum of **DST**.


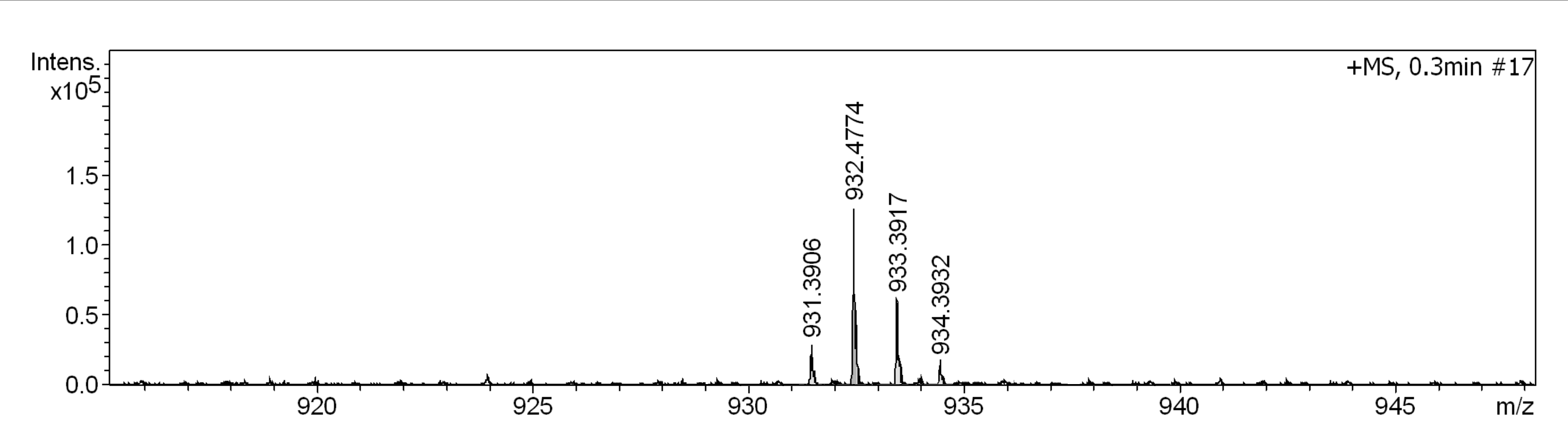


**Figure S9**. HR-MS spectrum of **DST**.

# Stabilities of DST probe toward determination of TYR and ATP.


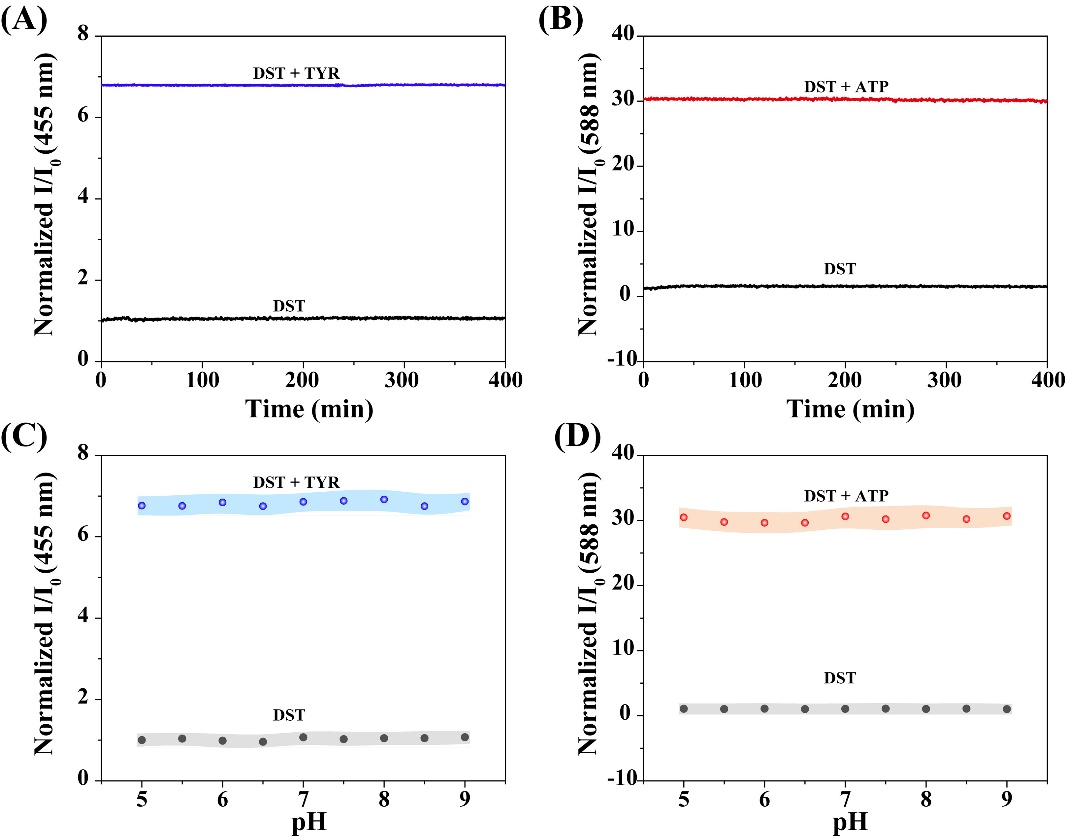


**Figure S10**. (A) Two-photon fluorescence stabilities of **DST** probe (5 μM) before (black line) and after (blue line) addition of TYR (20 U mL^-1^) (λ_ex_ = 720 nm, λ_em_ = 455 nm). (B) Two-photon fluorescence stabilities of **DST** probe (5 μM) before (black line) and after (red line) addition of ATP (30 mM) (λ_ex_ = 720 nm, λ_em_ = 588 nm). (C) pH stabilities of **DST** probe (5 μM) before and after addition of TYR (20 U mL^-1^). (D) pH stabilities of **DST** probe (5 μM) before and after addition of ATP (30 mM). Error bars show the SD for n=10.

# Competition tests of DST probe toward determination of TYR and ATP.


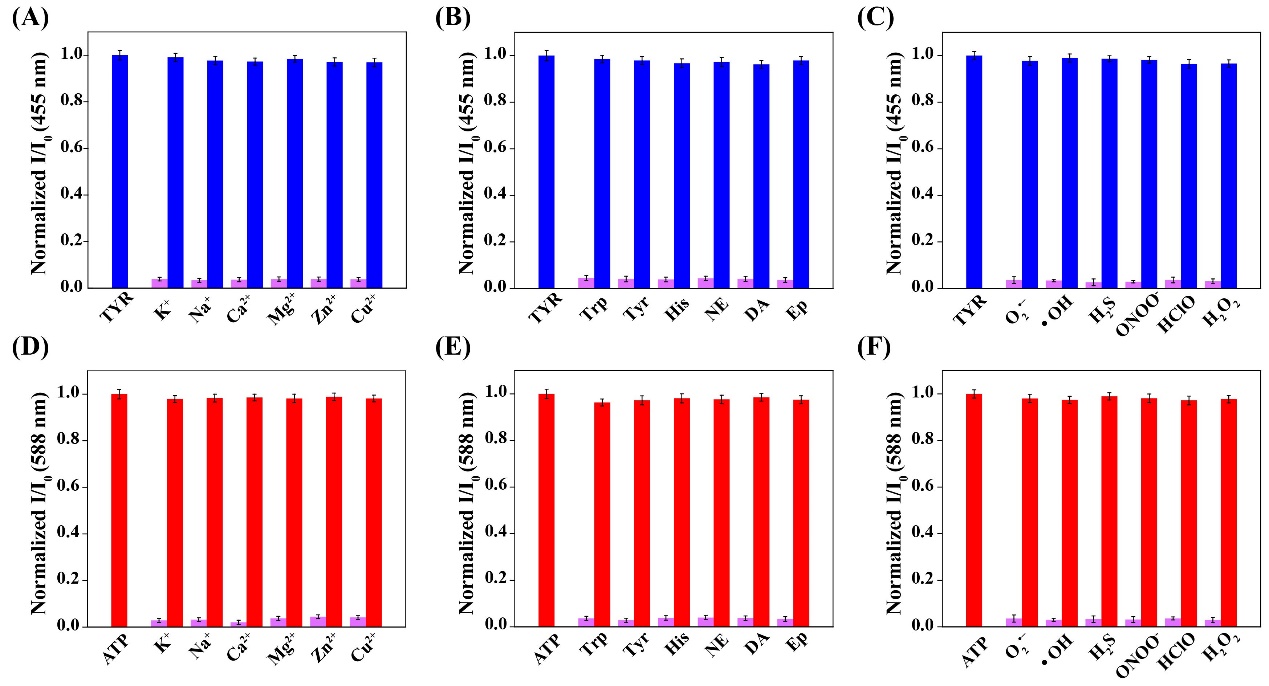


**Figure S11**. Competition tests of 5.0 μM **DST** probe toward TYR (A, B, and C) and ATP (D, E, and F) with the presence of (A and D) metal ions (1.0 mM for each), (B and E) proteins (20 U mL^-1^ for each), (C and F) ROS and other reactive substances (1.0 mM for each). Error bars show the SD for n=10.

# Sensing mechanism of DST probe to TYR and ATP.


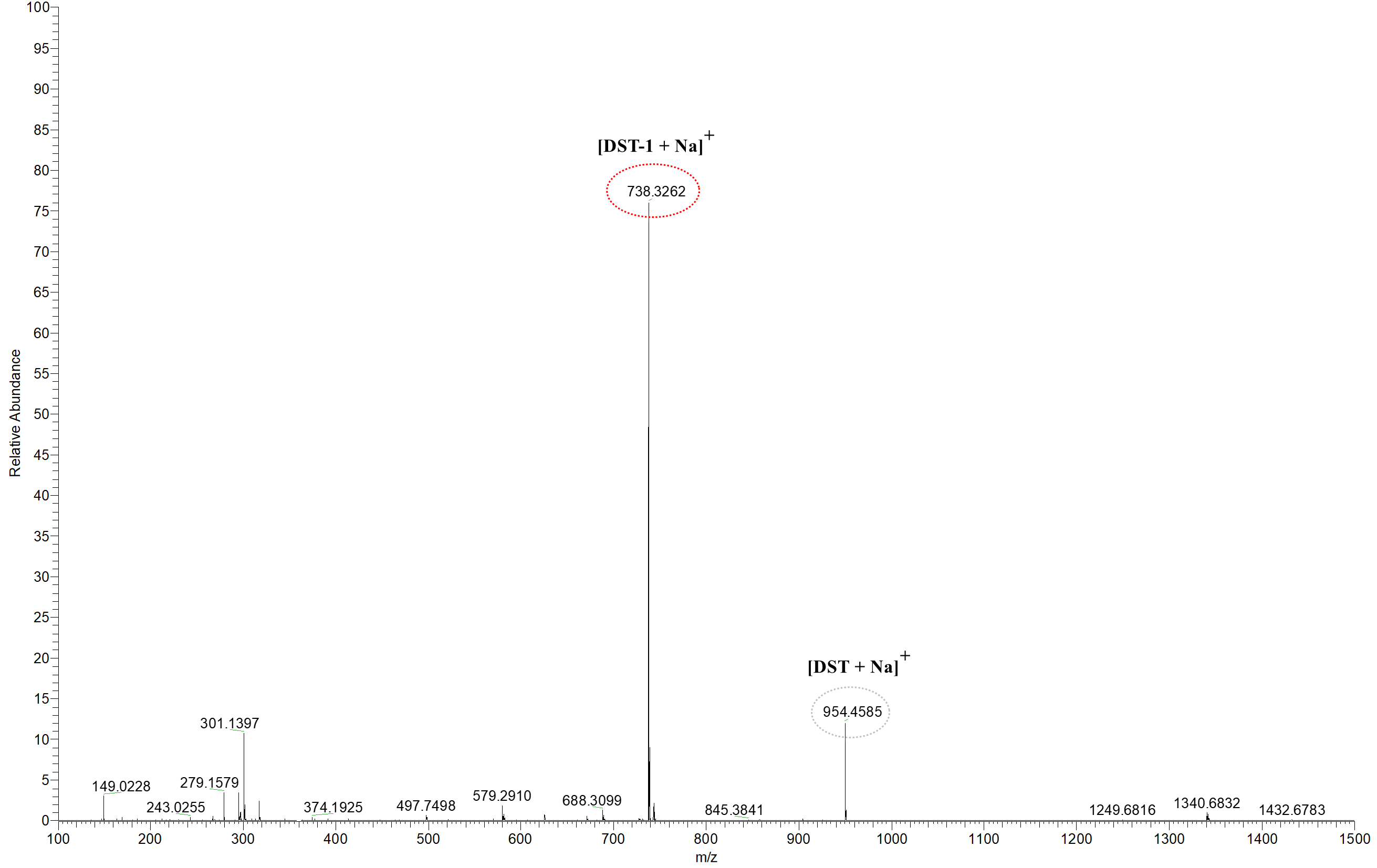


**Figure S12**. HR-MS of **DST** probe after interacted with TYR.

**
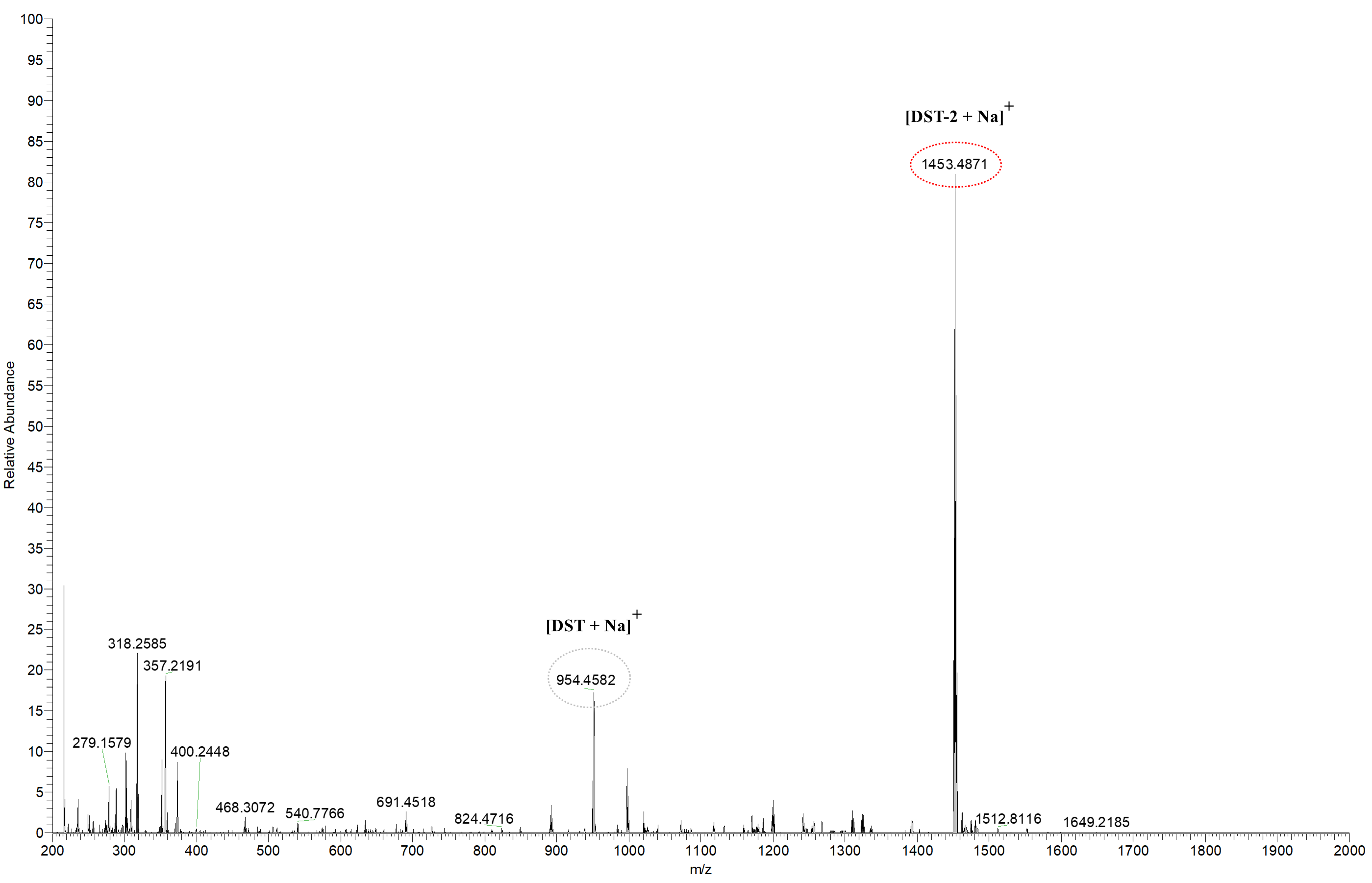
**

**Figure S13**. HR-MS of **DST** probe after interacted with ATP.

# FACS and MTT measurements of DST probe in live cells.

**
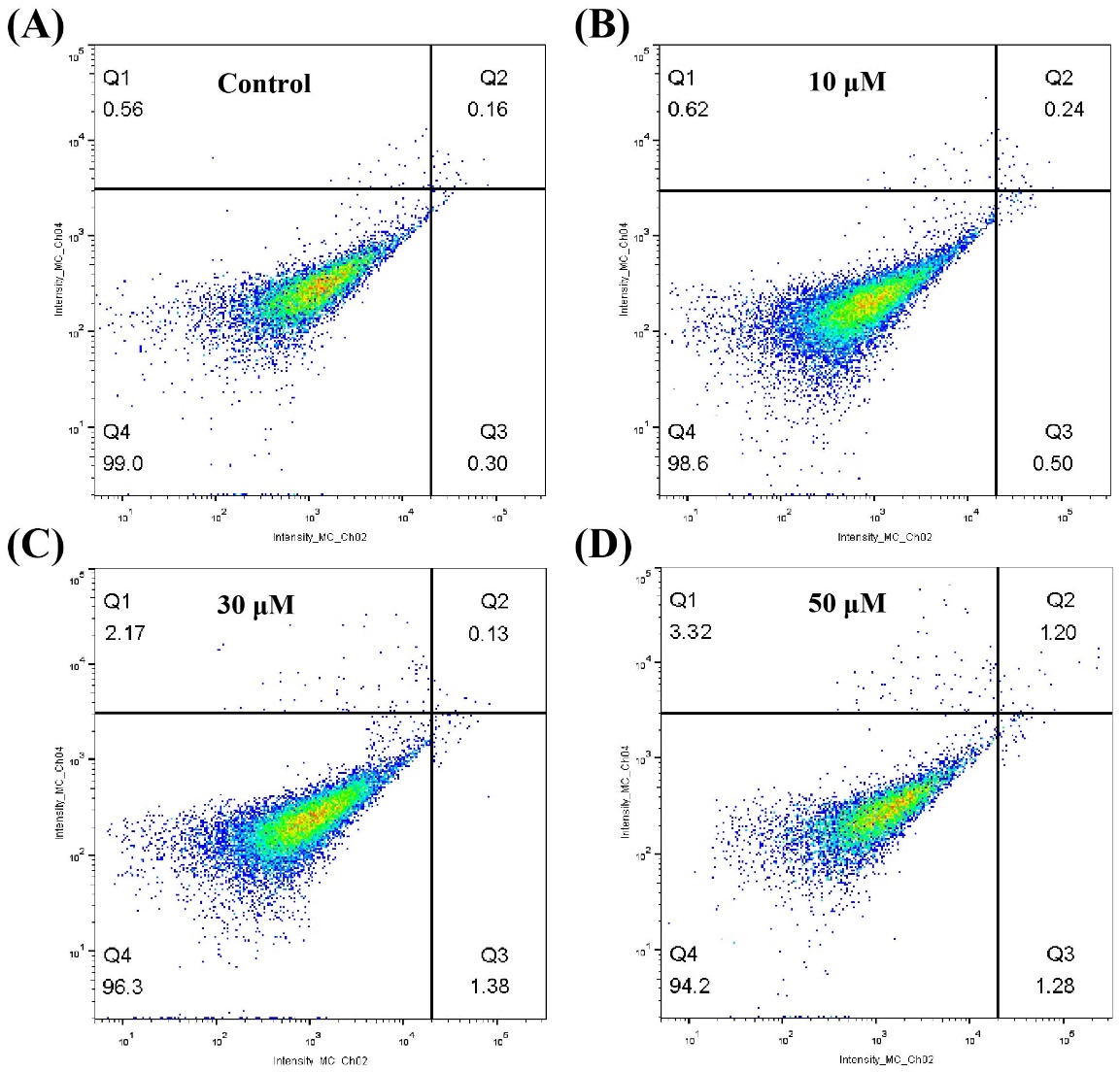
**

**Figure S14**. The apoptosis assay of neurons incubated with **DST** probe under different concentrations (A) 0 μM, (B) 10 μM, (C) 30 μM, and (D) 50 μM for 24 h. Q1, Q2, Q3, and Q4 represent the regions of dead neurons, late apoptotic neurons, early apoptotic neurons, and normal neurons, respectively.


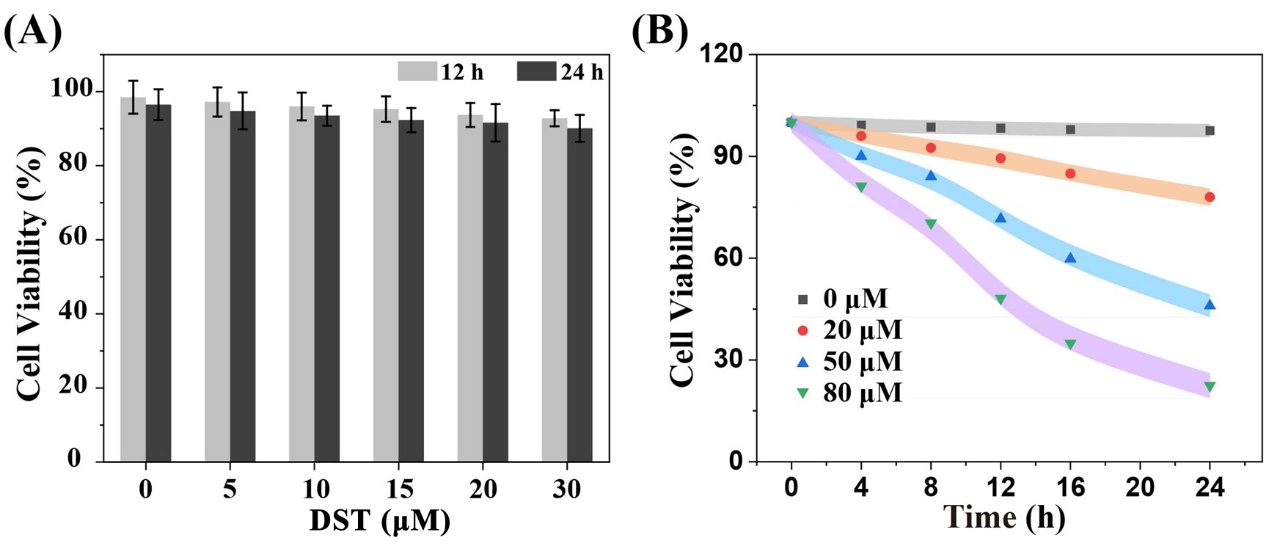


**Figure S15**. (A) The MTT assay for neurons upon incubation with **DST** at different concentrations (0, 5, 10, 15, 20, and 30 μM) after 12 h and 24 h, respectively. (B) Summarized data of neuron viability stimulated by various concentrations of O_2_**^•−^** (0, 20, 50, and 80 μM) for different times. Error bars show the SD for n=5.

# Western blot assay.


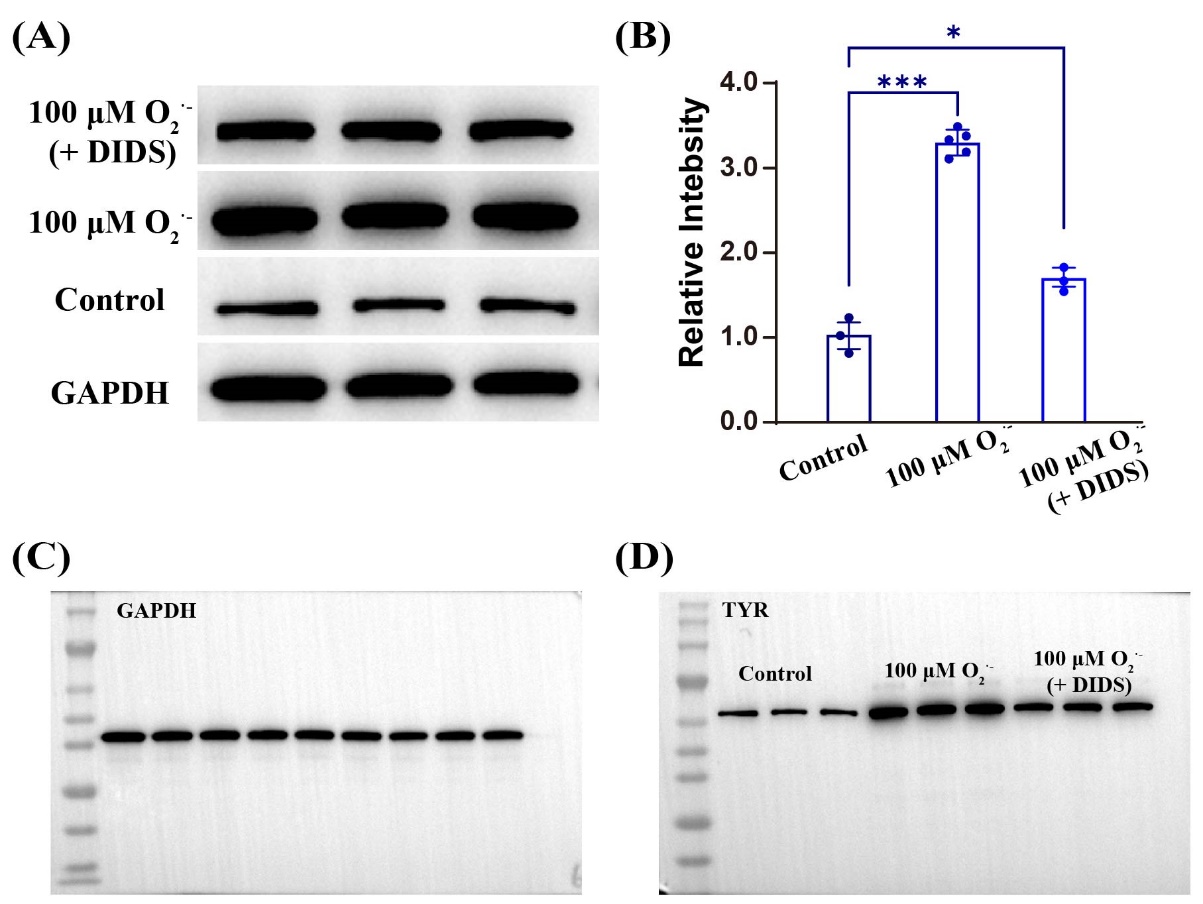


**Figure S16**. (A) Western blot analysis of TYR levels in neurons under different experimental conditions. TYR could be detected as protein bands of ~80 kDa and glyceraldehyde-3-phosphate dehydrogenase (GAPDH) was used as a protein standard (~37 kDa). (B) Relative expression of TYR in (A). The expression of TYR in normal conditions was denoted as 1.0. Asterisks indicate statistically significant changes (*P < 0.05, and ***P < 0.001). Error bars show the SD for n=3.

# Two-photon microscope imaging of TYR and ATP in response to O_2_^•−^ stimulation.


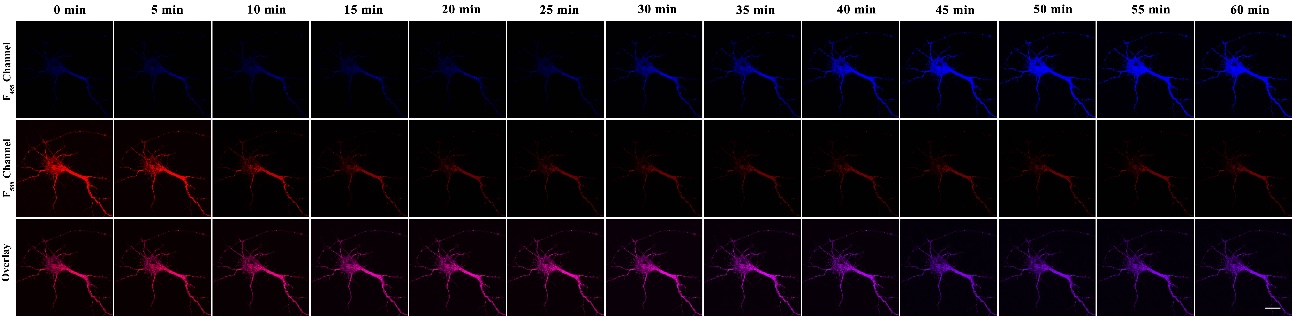


**Figure S17**. Confocal microscopy imaging of neurons after being treated with 100 μM O_2_^•−^ at different time points. Scale bar: 30 μm.


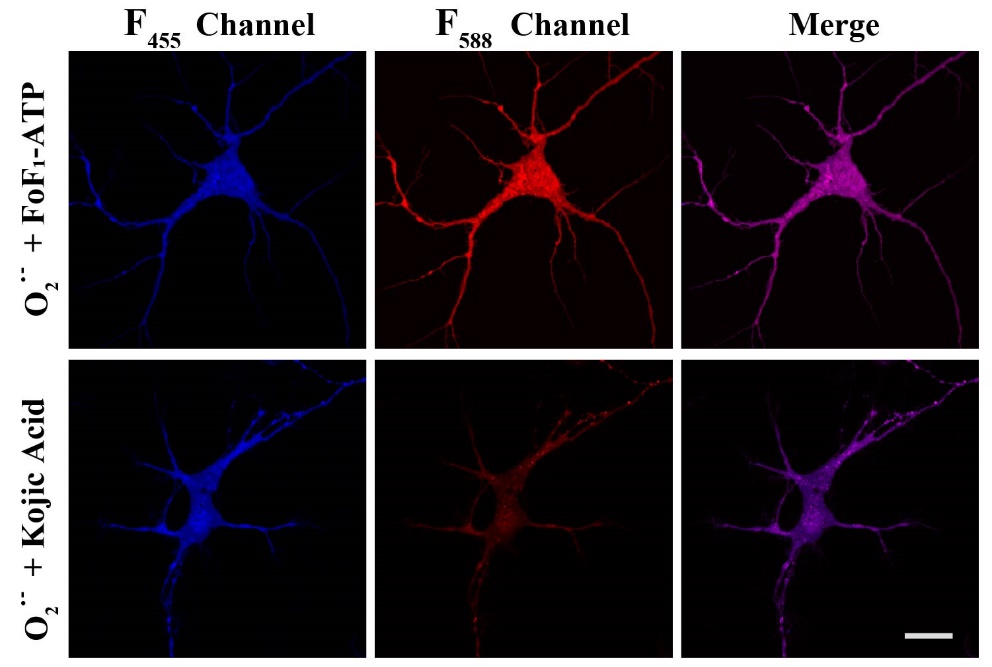


**Figure S18**. Confocal microscopy imaging of neurons after being treated with 100 μM O_2_^•−^ in the presence of 100 mg kg^-1^ kojic acid or 100 mg kg^-1^ FoF₁-ATP. Scale bar: 30 μm.
